# African swine fever virus hijacks lipolysis induced by chaperone-mediated autophagy to upregulate fatty acid β-oxidation and promote viral replication

**DOI:** 10.1128/mbio.03368-25

**Published:** 2026-03-09

**Authors:** Xing Yang, Xin Xiong, Huanan Liu, Xiaodan Wen, Xijuan Shi, Han Ma, Renpo Wang, Weijun Cao, Fan Yang, Yi Ru, Hong Tian, Jijun He, Jianhong Guo, Shichong Han, Zixiang Zhu, Haixue Zheng

**Affiliations:** 1 State Key Laboratory of Animal Disease Control and Prevention, College of Veterinary Medicine, Lanzhou University, Lanzhou Veterinary Research Institute, Chinese Academy of Agricultural Sciences, Lanzhou, China; 2 Gansu Province Research Center for Basic Disciplines of Pathogen Biology, Lanzhou, China; 3 International Joint Research Center of National Animal Immunology, College of Veterinary Medicine, Henan Agricultural University 731518, Zhengzhou, China; The University of Iowa, Iowa City, Iowa, USA

**Keywords:** African swine fever virus, fatty acid synthesis, fatty acid β-oxidation, lipid droplets, chaperone-mediated autophagy, lipolysis

## Abstract

Lipid metabolism plays a crucial role in cellular signal transduction, affects the structural integrity of cell membranes, and regulates energy metabolism. However, various viruses, including African swine fever virus (ASFV), usurp lipid metabolism to enhance their replication. The mechanism for the positive role of lipid metabolism in ASFV infection is unclear. Here, we present data that ASFV infection concurrently upregulates both fatty acid synthesis (FAS) and fatty acid β-oxidation (FAO) to enhance viral replication. Pharmacological inhibition of FAS significantly suppresses ASFV replication, an effect that can be markedly reversed by exogenous palmitate (the end product of FAS). Similarly, inhibition of FAO also impairs viral replication. Lipidomic profiling revealed that ASFV infection dramatically alters lipid droplet (LD) lipid composition, particularly triglycerides (TG) and diacylglycerols (DAG). ASFV infection triggers the accumulation of LDs, which in turn promote viral replication. Mechanistically, we discovered that ASFV exploits chaperone-mediated autophagy (CMA) to degrade perilipin 2 (PLIN2), a protein on the LD surface, thereby stimulating lipolysis. Furthermore, ASFV infection induces LD-mitochondrion contacts, facilitating the transfer of LD-derived fatty acids to mitochondria. These data indicate that LDs provide lipids to fuel ASFV-induced FAO upregulation. Collectively, our study reveals that ASFV orchestrates a complex metabolic network involving FAS, LD biogenesis, lipolysis, and FAO to optimize viral replication. These findings elucidate the pivotal role of lipid metabolism in ASFV replication, revealing a mechanism through which the virus manipulates cellular lipid pathways to facilitate its replication. This insight not only advances our understanding of ASFV pathogenesis but also presents potential therapeutic avenues for inhibiting viral production by modulating lipid metabolic processes.

**IMPORTANCE:**

African swine fever (ASF), caused by African swine fever virus (ASFV), represents a catastrophic threat to the global swine industry, with no safe and effective vaccines or antiviral therapies currently available except in Vietnam. Understanding how ASFV reprograms host lipid metabolism is critical for developing targeted interventions. Our study reveals a novel metabolic hijacking strategy employed by ASFV to reprogram lipid metabolism pathways, including fatty acid synthesis (FAS), lipid droplet (LD) biogenesis, chaperone-mediated autophagy (CMA)-mediated lipolysis, and mitochondrial β-oxidation (FAO), to support viral replication. Notably, we provide evidence that ASFV exploits CMA to degrade perilipin 2 (PLIN2), a key protein stabilizing lipid droplets, thereby promoting lipolysis. This mechanism resolves the paradox of concurrent upregulation of FAS and FAO by facilitating lipid shuttling through LD-mitochondrion contacts. Our findings offer new insights into how ASFV exploits host lipid networks and may pave the way for designing vaccines or targeted drugs to control ASF.

## INTRODUCTION

African swine fever virus (ASFV), a double-stranded nucleocytoplasmic large DNA virus, is a highly contagious and lethal pathogen that has caused devastating impacts on the global swine industry (1). ASFV infection causes mortality rates exceeding 90% in domestic pigs, posing a significant threat to economic stability and food security (2, 3). Emerging research highlights that ASFV hijacks host metabolic pathways to support viral replication, including lactate-mediated suppression of the innate immune response and amino acid depletion (4). Deciphering these host-pathogen metabolic interactions is crucial for developing targeted interventions.

Lipid metabolism, a central process governing cellular energy homeostasis and membrane biogenesis, encompasses pathways such as fatty acid synthesis (FAS), fatty acid β-oxidation (FAO), and the dynamic regulation of lipid droplets (LDs) (5, 6). Various viruses rely on the host lipid metabolism to ensure their normal replication cycle and promote their proliferation. For instance, FAS supplies lipid precursors for viral envelope assembly, as seen in hepatitis A virus (HAV), which enhances very long-chain fatty acid and sphingolipid synthesis to maintain its replication (7). Similarly, sterol regulatory element-binding protein (SREBP)-dependent lipogenesis is essential for the proliferation of Middle East respiratory syndrome coronavirus (MERS-CoV) and influenza A (H1N1) virus (8). FAO, which generates ATP via mitochondrial β-oxidation, is also manipulated by various viruses. Enterovirus A71 (EV71), dengue virus (DENV), and vaccinia virus all harness FAO-derived ATP to fuel their replication (9–11). In contrast, Japanese encephalitis virus (JEV) and human cytomegalovirus (HCMV) suppress FAO to retain cytoplasmic fatty acids for their own use (12, 13). Notably, FAS and FAO cooperatively regulate LD dynamics (14, 15).

LDs, the primary reservoirs of neutral lipids (e.g., triglycerides and cholesterol esters), are characterized by a hydrophobic core enveloped by a phospholipid monolayer studded with regulatory proteins such as perilipins (PLIN1-5) (16). LDs act as metabolic hubs, modulating lipid and energy homeostasis through coordinated FAS and FAO. Fatty acids synthesized via FAS are esterified into triglycerides (TAGs) for storage within LDs, while stored fatty acids can be released to fuel FAO-driven ATP production (17). Fatty acids are primarily liberated from LDs via two distinct mechanisms: lipolysis and lipophagy. In lipolysis, cytosolic lipases (e.g., ATGL and HSL) sequentially hydrolyze triglycerides (18). In lipophagy, an autophagic process, LDs are delivered to lysosomes for degradation (19). Recent studies have highlighted the role of chaperone-mediated autophagy (CMA) in regulating LD lipolysis (20), suggesting that CMA can be a potential target for antiviral therapies.

LDs are also associated with various viral infection processes. While LDs can contribute to antiviral immunity (21), several viruses manipulate LDs to promote viral replication in different ways. For example, rabies virus infection promotes LD formation and exploits LDs to facilitate budding by colocalizing viral proteins (M and G) with LDs (22). The nucleocapsid core of the hepatitis C virus (HCV) recruits the viral replication complex to LDs, with diacylglycerol acyltransferase 1 and 2 (DGAT1 and DGAT2) playing important roles in HCV assembly (23–25). Additionally, the SARS-CoV-2 ORF6 protein promotes LD lipolysis and mediates LD-mitochondrion interactions to produce ATP for viral replication (26). DENV infection induces LD accumulation and lipophagy, releasing fatty acids to upregulate FAO, which supports viral replication (27, 28). These studies underscore the essential roles of LDs in viral replication and pathogenesis. However, the role of LD in ASFV infection remains largely unexplored.

In this study, we investigated how ASFV reprograms host lipid metabolism. We demonstrate that ASFV concurrently upregulates FAS and FAO to enhance viral replication. Lipidomic analysis revealed that the most significantly altered metabolites following ASFV infection are closely associated with LDs. We further show that ASFV induces CMA to degrade PLIN2, thereby promoting lipolysis. The liberated fatty acids are subsequently shuttled from LDs to mitochondria through LD-mitochondrion contacts. These findings reveal a sophisticated lipid metabolic network orchestrated by ASFV, providing novel targets for antiviral development.

## RESULTS

### ASFV upregulates FAS to promote viral replication

**Fig 1:**
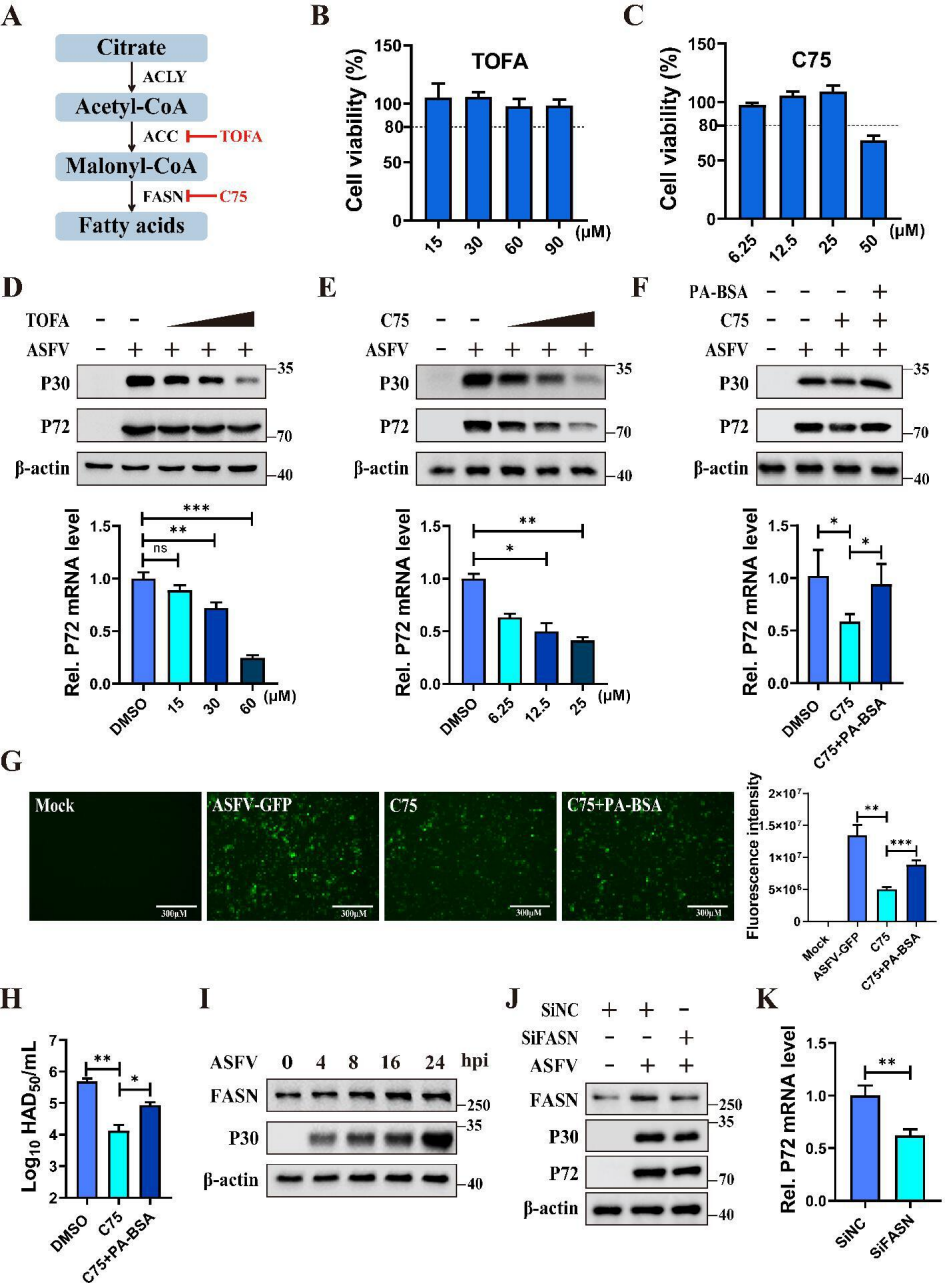
ASFV enhances FAS to promote viral replication. (**A**) Scheme of inhibitors employed to impede FAS pathways. (**B**) The effect of TOFA on cell viability. PAMs were treated with different concentrations of TOFA (15, 30, 60, and 90 µM) for 24 h, and cell viability was then analyzed. (**C**) The effect of C75 on cell viability. PAMs were treated with different concentrations of C75 (6.25, 12.5, 25 and 50 µM) for 24 h, and cell viability was analyzed. (**D**) Western blotting and RT-qPCR analysis of TOFA-mediated inhibition of ASFV. PAMs were treated with increasing concentrations of TOFA (15, 30, and 60 µM), and the same volume of DMSO was used as a negative control, followed by infection with ASFV (MOI = 0.1) for 24 h. Protein expression levels of ASFV p30 and p72, alongside p72 mRNA levels, were evaluated using western blotting and RT-qPCR. (**E**) Western blotting and RT-qPCR analysis of C75-mediated inhibition of ASFV. PAMs were treated with increasing concentrations of C75 (6.25, 12.5, and 25 µM) and the same volume of DMSO was used as a negative control, followed by infection with ASFV (MOI = 0.1) for 24 h. Protein levels of ASFV p30 and p72, along with p72 mRNA, were analyzed by western blotting and RT-qPCR. (**F–H**) PA-BSA partially restored the inhibition of ASFV replication by C75. PAMs were treated with C75 (25 µM) in conjunction with or without palmitic acid-BSA (50 µM) and infected with ASFV-WT or ASFV-GFP (MOI = 0.1) for 24 h; the same volume of DMSO was used as a negative control. Viral replication was then assessed through western blotting and RT-qPCR (**F**). GFP fluorescence was measured using a fluorescence microscope and quantified using Image J software (**G**). Scale bars = 300 µm. The viral titers were determined by virus titration (**H**). (**I**) ASFV infection increased FASN expression. Western blotting analysis of FASN, p30, and β-actin in PAMs infected with ASFV (MOI = 0.1) at 0, 4, 8, 16, and 24 hpi, respectively. (**J** and **K**) Knockdown of FASN inhibited ASFV replication. PAMs were transfected with siFASNs (siRNAs against *FASN* mRNA) for 36 h, followed by ASFV infection (MOI = 0.1) for an additional 24 h. Expression levels of FASN, p30, p72, and β-actin were subsequently detected by western blotting (**J**). The p72 mRNA levels were evaluated by RT-qPCR (**K**). Data are presented as the means ± SDs of three independent experiments and analyzed using Student’s *t*-test. *, *P* < 0.05; **, *P* < 0.01; ***, *P* < 0.001; ns, not significant.

FAS generates palmitate that is involved in protein palmitoylation, phospholipid synthesis, and energy production, and these processes are closely related to the viral life cycle (10). FAS is catalyzed by several key metabolic enzymes, including ATP citrate lyase (ACLY), acetyl-CoA carboxylase (ACC), and fatty acid synthase (FASN) (29). To assess whether FAS is required for ASFV replication, PAMs were treated with TOFA or C75, inhibitors of ACC and FASN, respectively (30) (Fig. 1A). Treatment with different concentrations of TOFA did not cause significant cytotoxicity in PAMs (Fig. 1B), and C75 showed no cytotoxic effects within the concentration range of 25 μM (Fig. 1C). PAMs treated with TOFA and infected with ASFV exhibited a dose-dependent inhibition of ASFV replication (Fig. 1D). Similarly, C75 also inhibited ASFV replication in a dose-dependent manner (Fig. 1E). However, the addition of exogenous palmitic acid-BSA (PA-BSA) to the culture in the presence of C75 significantly restored ASFV replication (Fig. 1F). Additionally, PA-BSA also restored the inhibition of ASFV-GFP replication by C75 (Fig. 1G). Virus titration exhibited the same results (Fig. 1H). These results confirm that FAS is critical for ASFV replication.

FASN is a key metabolic enzyme in fatty acid synthesis, which catalyzes successive condensation reactions to form fatty acids from malonyl-CoA and acetyl-CoA substrates, primarily producing 16-carbon palmitate (31). To investigate the role of FASN in ASFV infection, PAMs were mock-infected or infected with ASFV (multiplicity of infection [MOI] = 0.1) at different time points (0, 4, 8, 16, and 24 h). Western blotting analysis revealed that FASN protein expression gradually increased as the infection progressed (Fig. 1I). To further determine the role of FASN in ASFV infection, PAMs were transfected with FASN-specific small interfering RNAs (siRNAs), followed by ASFV infection. Western blotting and RT-qPCR analysis indicated that knockdown of FASN significantly decreased ASFV replication (Fig. 1J and K), highlighting the critical role of FASN in ASFV replication. To investigate which step of the ASFV life cycle is affected by FAS, we analyzed the effect of C75 inhibitor on binding and entry into PAMs; pretreatment of PAMs with C75 did not lead to a decrease in ASFV binding and entry (Fig. S1A and B). To further understand its mechanism of action, we performed a time-of-addition experiment by adding 25 μM C75 to PAMs before or during ASFV infection. A significant reduction in viral genome copy number was observed at different time points when C75 was added (Fig. S1C). These data indicate that the upregulated FAS is indispensable for productive ASFV replication post-entry. Taken together, these findings demonstrate that FAS is a critical pathway hijacked by ASFV to support its replication.

### Fatty acid β-oxidation is required for ASFV replication

**Fig 2:**
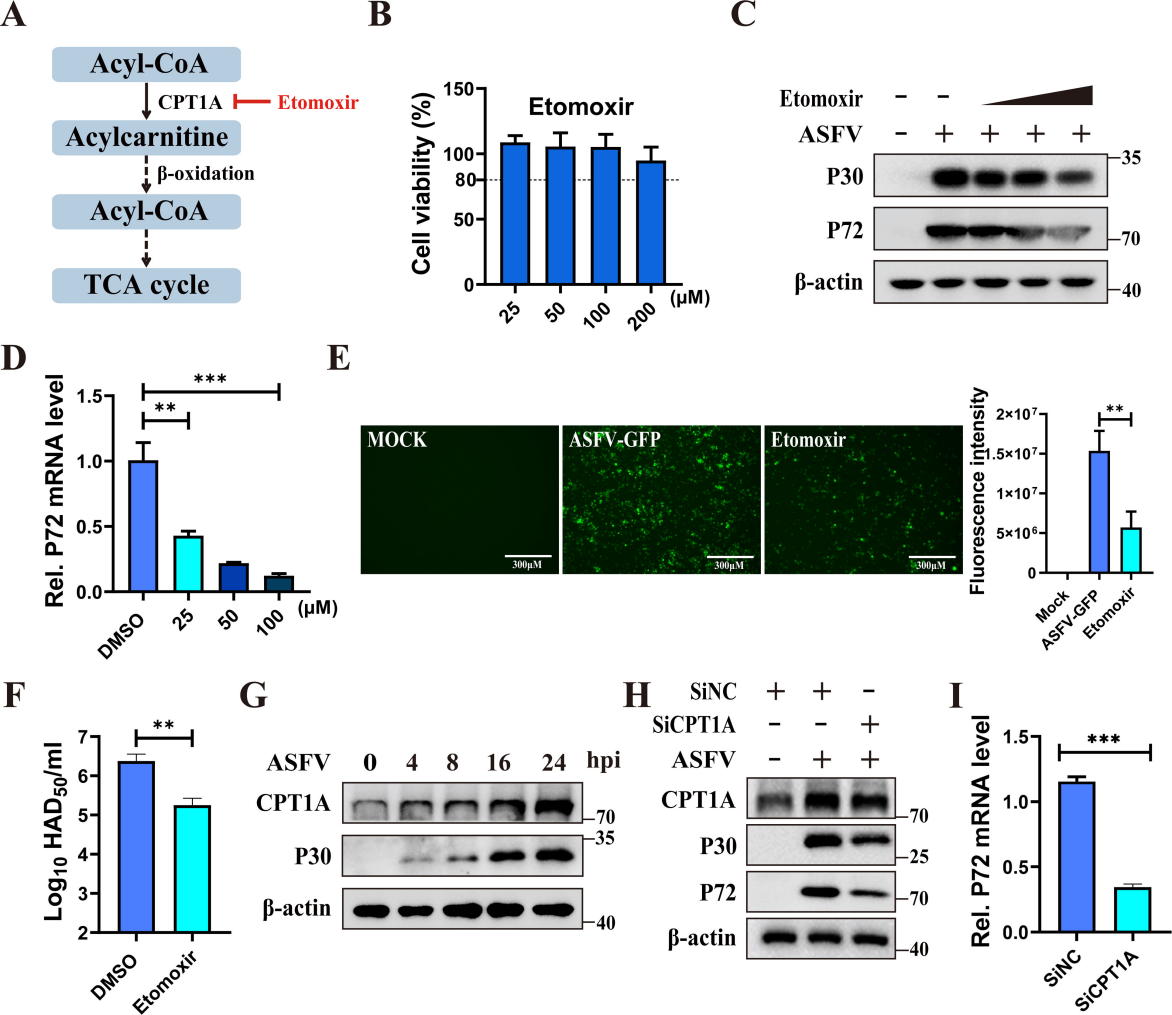
ASFV promotes viral replication through exploiting fatty acid β-oxidation. (**A**) Scheme of an inhibitor employed to inhibit fatty acid β-oxidation. (**B**) The effect of etomoxir on cell viability. PAMs were treated with different concentrations of etomoxir (25, 50, 100, and 200 µM) for 24 h, and cell viability was subsequently assessed. (**C–F**) Inhibition of fatty acid β-oxidation inhibits ASFV replication. PAMs were treated with increasing concentrations of etomoxir (25, 50, and 100 µM), and the same volume of DMSO was used as a negative control, followed by infection with ASFV (MOI = 0.1). Western blotting was performed to determine the expression levels of p30, p72, and β-actin (**C**), while RT-qPCR was employed to analyze p72 mRNA levels (**D**). PAMs were treated with etomoxir (100 µM) for 24 h and then infected with ASFV-GFP (MOI = 0.1); the GFP expression was captured by a fluorescence microscope, and GFP fluorescence intensity was quantified using Image J software (**E**). Scale bars = 300 µm. The viral titers were assessed by hemadsorption (HAD) assay (**F**). (**G**) ASFV infection upregulated CPT1A expression. Western blotting analysis of CPT1A, p30, and β-actin in PAMs infected with ASFV (MOI = 0.1) for 0, 4, 8, 16, and 24 h, respectively. (**H and I**) Knockdown of CPT1A inhibited ASFV replication. PAMs were transfected with SiCPT1As for 36 h, followed by ASFV infection at an MOI of 0.1 for an additional 24 h. Viral replication was detected by western blotting (**H**) and RT-qPCR (**I**). Data are presented as the means ± SDs of three independent experiments and analyzed using Student’s *t*-test. **, *P* < 0.01; ***, *P* < 0.001.

Mitochondria are the principal sites for FAO. Carnitine palmitoyltransferase 1A (CPT1A) is the key rate-limiting enzyme of FAO, responsible for transporting fatty acids from the cytoplasm to the mitochondria (32). To investigate the role of FAO in ASFV infection, we selected etomoxir, an inhibitor of CPT1A, to inhibit FAO and evaluated the viral replication in the presence of etomoxir (Fig. 2A). Treatment with different concentrations of etomoxir did not cause significant cytotoxicity to PAMs (Fig. 2B). Etomoxir treatment considerably inhibited ASFV replication in a dose-dependent manner in PAMs (Fig. 2C and D), consistant with our previous research results(33). Additionally, it also decreased ASFV-GFP replication (Fig. 2E), and virus titration confirmed that etomoxir significantly inhibited the production of progeny ASFV (Fig. 2F). These results demonstrate that FAO is essential for ASFV replication. Subsequently, PAMs infected with ASFV (MOI = 0.1) were collected at the indicated time points, and the CPT1A protein level was analyzed. Western blotting showed that CPT1A protein expression was gradually upregulated as the infection progressed (Fig. 2G). To determine the role of FAO in ASFV infection, PAMs were transfected with CPT1A-specific siRNA, followed by ASFV infection. Knockdown of CPT1A significantly decreased the replication of ASFV (Fig. 2H and 2I). These findings demonstrate that ASFV exploits FAO to support its replication.

### ASFV infection significantly alters LD-related metabolism in PAMs

**Fig 3:**
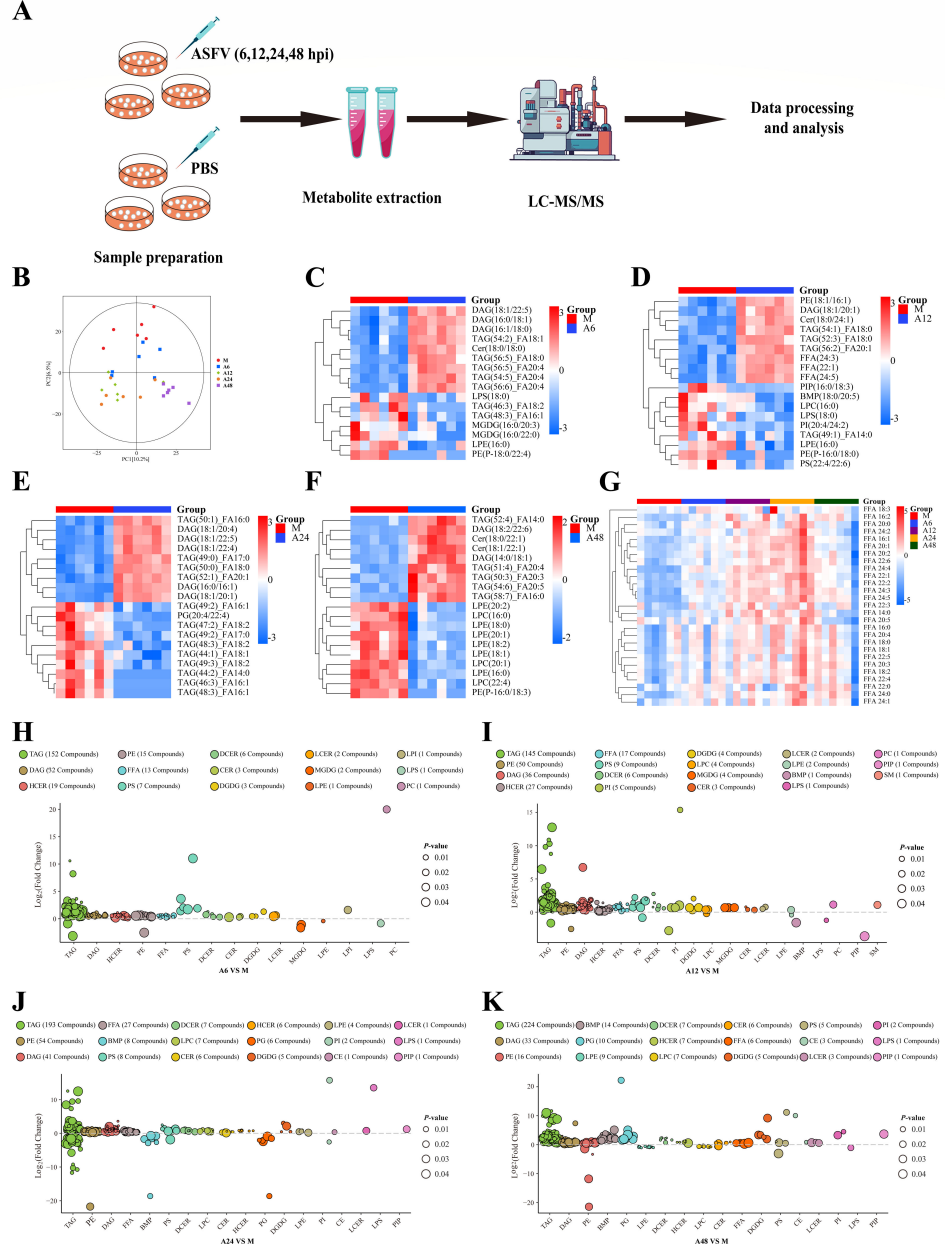
Lipidomic profiling analysis of the ASFV-infected PAMs. (**A**) Schematic representation of the lipidomic analysis workflow for the ASFV-infected PAMs. (**B**) PCA of PAMs infected with ASFV at 6, 12, 24, and 48 hpi or mock-infected, with six biological replicates per condition; each point represents one biological replicate. (**C–F**) Heat map of hierarchical clustering analysis of differential metabolites after ASFV infection. The top 10 most significantly upregulated or decreased metabolites were exhibited. Each column represents one sample, and each row represents one differential metabolite. Red indicates upregulation, while blue signifies downregulation. (**G**) Heat map of hierarchical clustering analysis of differential fatty acids during ASFV infection. Red indicates upregulation, while blue signifies downregulation. (**H**) Species analysis of lipid metabolites differentially altered in ASFV-infected PAMs at 6 h (A6) relative to mock (M) infection. (**I**) Species analysis of lipid metabolites differentially altered in ASFV-infected PAMs at 12 h (A12) relative to mock (M) infection. (**J**) Species analysis of lipid metabolites differentially altered in ASFV-infected PAMs at 24 h (A24) relative to mock (M) infection. (**K**) Species analysis of lipid metabolites differentially altered in ASFV-infected PAMs at 48 h (A48) relative to mock (M) infection. Data points are means from six biological replicates; each data point represents a lipid metabolite. Log_2_(fold change) relative to mock infection is shown on the *x*-axis. Individual lipid species are colored by the class of lipid to which they belong. DAG, diacylglycerol; TAG, triacylglycerol; FFA, free fatty acids; PC, phosphatidylcholine; PE, phosphatidylethanolamine; PG, phosphatidylglycerol; PI, phosphatidylinositol; PS, phosphatidylserine; MGDG, monogalactosyldiacylglycerol; DGDG, digalactosyldiacylglycerol; CE, cholesterol esters; CER, ceramides; DCER, dihydroceramides; HCER, hexosylceramides; LCER, lactosylceramides; SM, sphingomyelins; LPC, lysophosphatidylcholines; LPE, lysophosphatidylethanolamines; LPG, lyso-phosphatidylglycerol; LPI, lyso-phosphatidylinositol; LPS, lyso-phosphatidylserine; PIP, phosphatidylinositol phosphate; and BMP, bis(monoacylglycero)phosphate.

FAS and FAO are reciprocally regulated under normal physiological conditions. Malonyl-CoA, a substrate for *de novo* lipogenesis, can inhibit CPT1A-mediated FAO (34, 35). However, we confirmed that ASFV concurrently upregulates both FAS and FAO in PAMs. LDs are crucial organelles for maintaining lipid homeostasis in cells. We hypothesized that LDs may play a role in maintaining lipid balance during ASFV infection. PAMs were infected with ASFV and collected at 6, 12, 24, and 48 hpi (A6, A12, A24, and A48). Global lipidomic profiling of the ASFV-infected PAMs at these time points was performed using LC-MS/MS analysis (Fig. 3A). Principal component analysis (PCA) confirmed that the infection status accounted for most of the changes, with ASFV-infected samples and mock samples forming distinct clusters (Fig. 3B). Lipidomics revealed significant alterations in lipid metabolites: 258 metabolites (251 increased and 7 decreased) between A6 and mock infection, 303 metabolites (294 increased and 9 decreased) between A12 and mock infection, 378 metabolites (326 increased and 52 decreased) between A24 and mock infection, and 364 metabolites (338 increased and 26 decreased) between A48 and mock infection (*P* < 0.05 and VIP > 1) (Table S1). ASFV infection induced significant upregulation of numerous lipid metabolites (Fig. S2). Analysis of the top 10 most altered lipid metabolites following ASFV infection revealed significant changes in triglycerides (TAGs) and diacylglycerols (DAGs), key components of LDs (Fig. 3C through F). TAGs are the core components of LDs, and DAGs are key intermediates in TAG synthesis and breakdown. These results suggest that LD-related metabolism is closely related to ASFV replication. Furthermore, we investigated the changes in free fatty acid (FFA) during ASFV infection and found that FFAs were dynamically changed during ASFV infection. While FFA levels were significantly elevated before 24 hpi compared to the mock control, they exhibited a notable decrease at 48 hpi relative to the 24 hpi (Fig. 3G). The decrease of FFAs at 48 hpi likely reflects the rapid consumption of these FFAs to fuel the viral replication. These results indicate that LDs may serve as central hubs in ASFV-induced reprogramming of lipid metabolism. Moreover, analysis of the species of differentially changed lipids after ASFV infection has shown that structural and envelope lipids such as phosphatidylethanolamine (PE), phosphatidylserine (PS), and phosphatidylglycerol (PG) increased significantly, and signaling lipids (dihydroceramides [DCER], hexosylceramides [HCER], and ceramides [CER]) also increased (Fig. 3H through K). Taken together, these data suggest that ASFV reprograms host lipid metabolism to meet virion assembly and energy requirements, creating a favorable environment for virus replication.

### ASFV infection induces LD biogenesis

**Fig 4:**
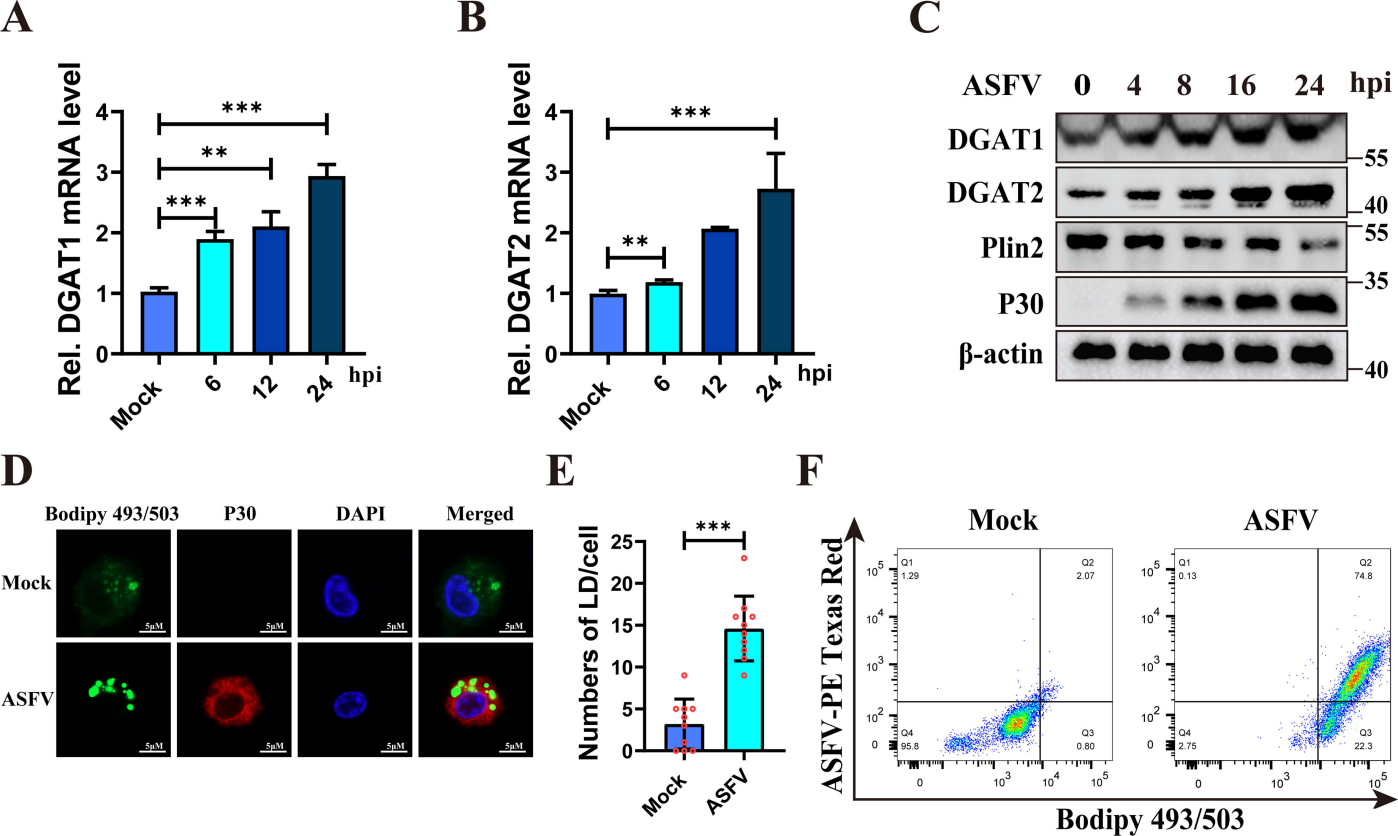
ASFV infection induces LD biogenesis while reducing PLIN2 levels. (**A and B**) ASFV infection increased the transcription of DGAT1 and DGAT2 in PAMs. PAMs were infected with ASFV (MOI = 0.1) for 0, 6, 12, and 24 h. The mRNA levels of DGAT1 (**A**) and DGAT2 (**B**) were quantified using RT-qPCR, respectively. (**C**) ASFV infection increased the expression of DGAT1 and DGAT2 in PAMs. PAMs were infected with ASFV (MOI = 0.1) for 0, 4, 8, 16, and 24 h. Expression of DGAT1, DGAT2, PLIN2, p30, and β-actin was analyzed by western blotting. (**D and E**) ASFV infection increased the number of LD in PAMs. PAMs were mock-infected or infected with ASFV (MOI = 0.1) for 24 h. ASFV p30 was detected by immunofluorescence, and LDs were visualized using BODIPY493/503 staining (**D**). Scale bars = 5 µm. Quantification of LD numbers per cell from (**D**) using the Image J software (*n* = 10) (**E**). (**F**) Flow cytometric analysis of LDs in the ASFV-infected PAMs. PAMs were mock-infected or infected with ASFV-RFP (MOI = 0.1) for 24 h. Intracellular LDs in PAMs were measured using Bodipy 493/503 staining, followed by flow cytometric analysis at 24 hpi. Data are presented as the means ± SDs of three independent experiments and analyzed using Student’s *t*-test. **, *P* < 0.01; ***, *P* < 0.001.

LD biogenesis originates in the endoplasmic reticulum, with DGAT1 and DGAT2 being critical enzymes for LD formation (36). To verify the changes in LDs following ASFV infection, we measured the mRNA levels of DGAT1 and DGAT2. The results showed that the mRNA levels of both enzymes increased in a time-dependent manner after ASFV infection (Fig. 4A and B). Concurrently, the protein levels of DGAT1 and DGAT2 were upregulated (Fig. 4C), indicating that ASFV infection promotes LD biogenesis. Interestingly, PLIN2, a surface protein of LDs that protects LD from lipolysis (37, 38), was downregulated during ASFV infection (Fig. 4C). More importantly, the expression of PLIN2 was significantly reduced, while FASN and CPT1A were significantly increased in the lung and spleen of ASFV-infected pigs compared to uninfected controls, consistent with the above results in ASFV-infected PAMs (Fig. S3). Staining with Bodipy 493/503 revealed a significant accumulation of LDs in ASFV-infected PAMs, as evidenced by an increase in LD number (Fig. 4D and E). Flow cytometry also demonstrated an increase in LDs in the ASFV-infected cells (Fig. 4F). These results demonstrate that ASFV infection enhances LD biogenesis.

### LD accumulation promotes ASFV replication

**Fig 5:**
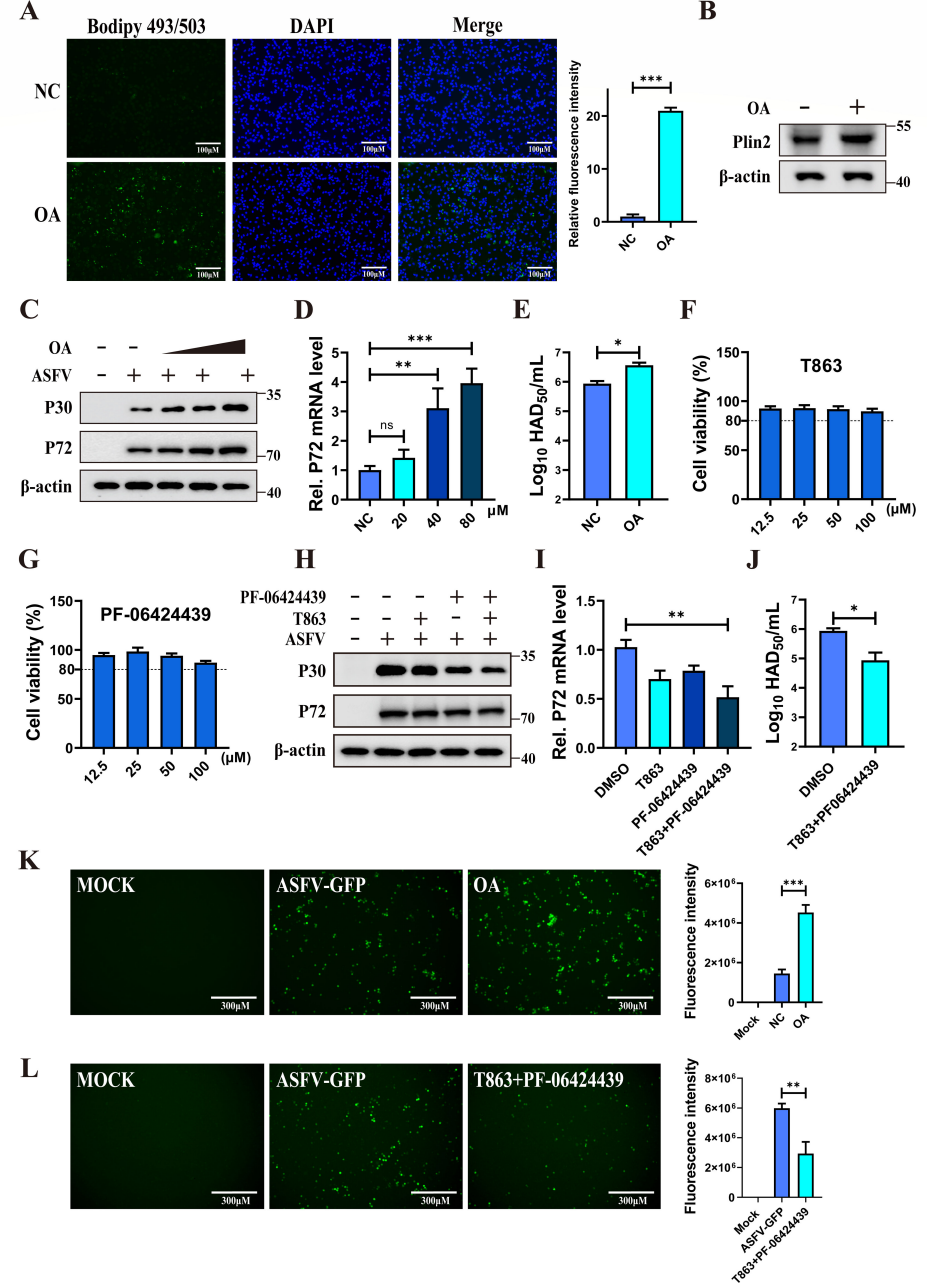
LDs facilitate ASFV replication. (**A and B**) OA induced LD biogenesis in PAMs. PAMs were treated with OA (80 µM) for 24 h, and the same volume of the solvent was added as a control. LDs were stained with Bodipy 493/503 (green), nucleus was stained with DAPI (blue), and the images were captured by a fluorescence microscope. The fluorescence intensity of LDs was quantified using Image J software (**A**). The protein levels of PLIN2 and β-actin were analyzed by western blotting (**B**). (**C and D**) OA promotes ASFV replication. PAMs were treated with increasing concentrations of OA (0, 20, 40, and 80 µM) for 24 h, followed by infection with ASFV (MOI = 0.1) for an additional 24 h. Western blotting was then performed to determine the expression levels of p30, p72, and β-actin (**C**), while RT-qPCR was employed to analyze p72 mRNA levels (**D**). (**E**) OA enhanced viral titers of ASFV in PAMs. PAMs were treated with OA (80 µM) for 24 h and infected with ASFV (MOI = 0.1) for another 24 h. The viral titers were determined by the HAD assay. (**F and G**) The effect of T863 and PF-06424439 on cell viability. PAMs were treated with different concentrations of PF-06424439 (12.5, 25, 50, and 100 µM) and T863 (12.5, 25, 50 and 100 µM) for 24 h, and cell viability was analyzed through the CCK-8 assay. (H–J) T863 and PF-06424439 inhibited ASFV replication on PAMs. PAMs were treated with PF-06424439 (50 µM) and/or T863 (50 µM) and infected with ASFV (MOI = 0.1) for 24 h. The same volume of DMSO was added as a negative control. Viral replication was determined by western blotting (**H**) and RT-qPCR (**I**). The viral titers of PF-06424439 (50 µM) and T863 (50 µM) combined were determined by the HAD assay (**J**). (**K**) Effect of OA on ASFV-GFP replication in PAMs. PAMs were treated with OA (80 µM) or the same volume of the solvent for 24 h, followed infection with ASFV-GFP (MOI = 0.1). Images were captured under a fluorescence microscope, and the GFP fluorescence intensity was quantified using Image J software. Scale bars = 300 µm. (**L**) Effect of DGAT inhibitors on ASFV replication in PAMs. PAMs were treated with PF-06424439 (50 µM) and T863 (50 µM) for 24 h, and DMSO was used as a blank control. The samples were mock-infected and ASFV-infected (MOI = 0.1) for another 24 h, and the images were captured under a fluorescence microscope. The GFP fluorescence intensity was quantified using Image J software. Scale bars = 300 µm. Data are presented as the means ± SDs of three independent experiments and analyzed using Student’s *t*-test. *, *P* < 0.05; **, *P* < 0.01; ***, *P* < 0.001.

To elucidate the role of LDs during ASFV infection, oleic acid (OA) was used to induce LD biogenesis in PAMs. OA treatment led to LD accumulation and an increase in PLIN2 protein expression in PAMs (Fig. 5A and B). PAMs were then treated with increasing concentrations of OA for 12 h, followed by ASFV infection for another 24 h. Western blotting and RT-qPCR analyses showed that OA promoted ASFV replication in a dose-dependent manner (Fig. 5C and D). Virus titration also showed that OA treatment increased the viral titer of ASFV in PAMs (Fig. 5E). These data demonstrated that OA-induced LD biogenesis enhances ASFV replication. To further investigate this effect, PF-06424439 (DGAT1 inhibitor) and T863 (DGAT2 inhibitor) were employed to inhibit the LD biogenesis. Treatment with these inhibitors at various concentrations did not cause significant cytotoxicity in PAMs (Fig. 5F and G). PAMs were treated with DGAT1 and/or DGAT2 inhibitors and subsequently infected with ASFV. As expected, inhibition of either DGAT1 or DGAT2 decreased viral replication, with the combined inhibition of both enzymes having the most pronounced effect on ASFV replication (Fig. 5H through J). Fluorescence results also showed that OA promoted ASFV-GFP replication, whereas DGAT1 and DGAT2 inhibitors decreased ASFV replication (Fig. 5K and L). Additionally, inhibition of DGAT1 and/or DGAT2 attenuated the OA-induced enhancement of ASFV replication (Fig. S4). Together, these findings indicate that LDs promote ASFV replication.

### CMA-mediated degradation of PLIN2 promotes lipolysis during ASFV infection

**Fig 6:**
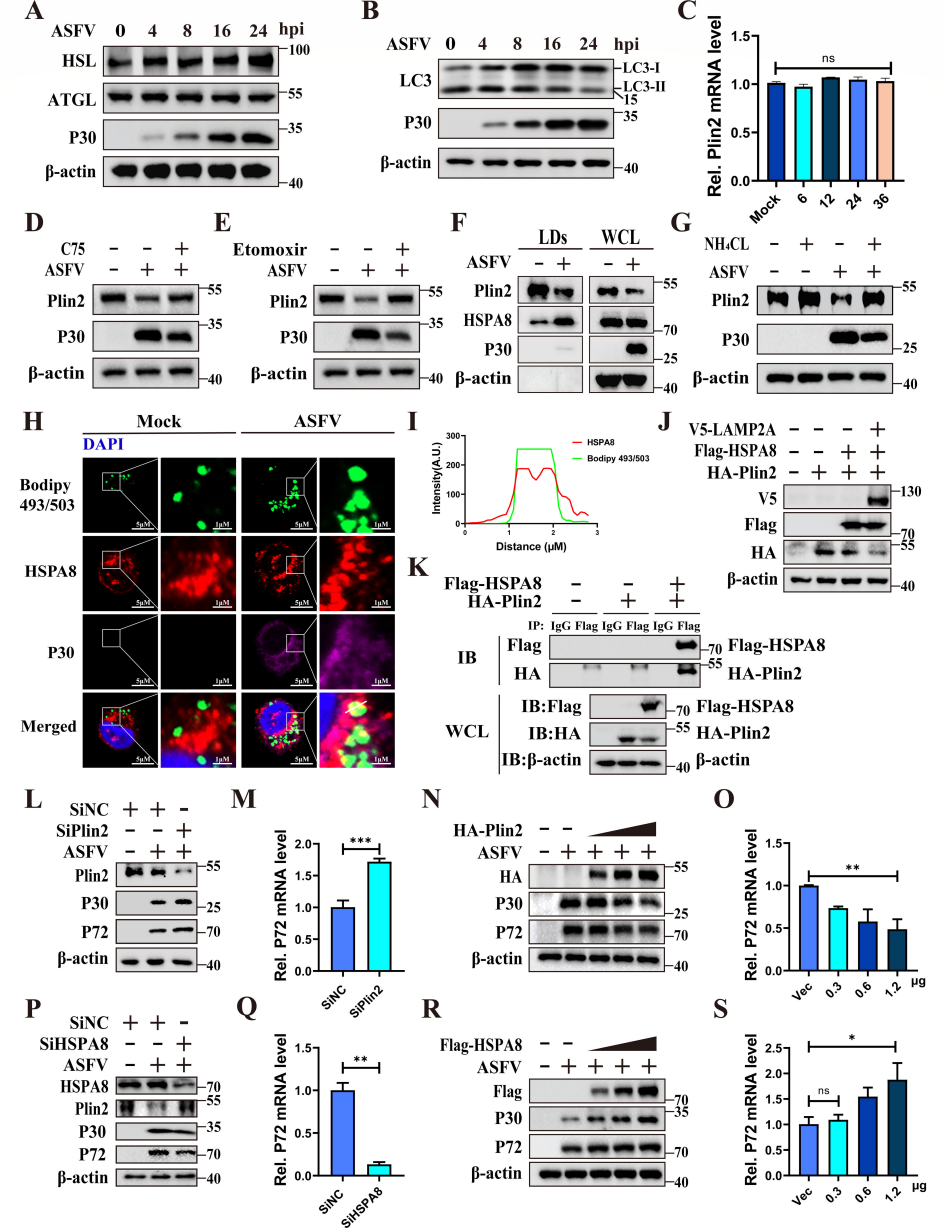
ASFV degrades PLIN2 through CMA to promote lipolysis. (**A**) ASFV infection upregulated the expression of HSL, but ATGL has not changed. PAMs were infected with ASFV for 0, 4, 8, 16, and 24 h, and the protein levels of ATGL, HSL, p30, and β-actin were determined by western blotting. (**B**) ASFV infection upregulated LC3-I and downregulated LC3-II at the middle and late stages in PAMs. PAMs were infected with ASFV (MOI = 0.1) for 0, 4, 8, 16, and 24 h, and the protein levels of LC3, p30, and β-actin were determined by western blotting. (**C**) The mRNA level of PLIN2 has no change during ASFV infection. PAMs were infected with ASFV (MOI = 0.1) for 0, 6, 12, 24, and 36 h, and the mRNA level of PLIN2 was detected by RT-qPCR. (**D and E**) Inhibition of ASFV replication inhibited PLIN2 degradation. PAMs were treated with C75 (**D**) or etomoxir (**E**) and infected with ASFV (MOI=0.1) for 24 h, and the protein levels of PLIN2, p30, and β-actin were determined by western blotting. (**F**) HSPA8 enrichment in purified LDs from the ASFV-infected PAMs. PAMs were infected with ASFV (MOI = 1) and mock-infected for 24 h. Then, LDs were purified from these cells, and the protein levels of PLIN2, HSPA8, p30, and β-actin were analyzed by western blotting. (**G**) NH_4_Cl restored the degradation of PLIN2 during ASFV infection. PAMs were infected with ASFV (MOI = 0.1) for 8 h and then treated with NH_4_Cl (10 mM) for 16 h. The protein levels of PLIN2, p30, and β-actin were analyzed by western blotting. (**H and I**) HSPA8 recruitment to LDs upon ASFV infection. PAMs were infected with ASFV (MOI = 0.1) or mock-infected for 24 h. ASFV-p30 (purple) and HSPA8 (red) were detected by immunofluorescence analysis, and LDs were examined by Bodipy 493/503 staining (green) (**H**). Scale bars, 5 μm. Intensity within the indicated area was measured (**I**). (**J**) HSPA8 and LAMP2A decreased the expression of PLIN2. HEK-293T cells were transfected with HA-PLIN2, Flag-HSPA8, and V5-LAMP2A plasmids for 24 h, and the expression levels of HA-PLIN2, Flag-HSPA8, and V5-LAMP2A were determined by western blotting. (**K**) HSPA8 interacts with PLIN2. HEK293T cells were co-transfected with Flag-HSPA8 and HA-PLIN2, and pCDNA3.1 was used as a negative control. At 24 h post-transfection, the lysates were collected and incubated with anti-Flag beads, and then, the bound proteins were examined by western blotting. (**L and M**) Knockdown of PLIN2 promotes ASFV replication. PAMs were transfected with siPLIN2s for 36 h, followed by ASFV infection (MOI = 0.1) for an additional 24 h. Expression levels of PLIN2, p30, p72, and β-actin were subsequently detected by western blotting (**L**). The p72 mRNA levels were evaluated using RT-qPCR (**M**). (**N and O**) Exogenous expression of PLIN2 inhibits ASFV replication. WSL cells were transfected with an increasing dose of HA-PLIN2 plasmid for 24 h, and then infected with ASFV (MOI = 1) for 24 h. Viral replication was analyzed by western blotting (**N**) and RT-qPCR (O). (**P and Q**) Knockdown of HSPA8 partially restored the expression of PLIN2 and inhibited ASFV replication. PAMs were transfected with siHSPA8s (siRNAs against HSPA8 mRNA) for 36 h, followed by ASFV infection (MOI = 0.1) for an additional 24 h. Expression levels of HSPA8, PLIN2, p30, p72, and β-actin were subsequently detected by western blotting (**P**). The p72 mRNA levels were evaluated by RT-qPCR (**Q**). (**R and S**) Exogenous expression of HSPA8 promotes ASFV replication. WSL cells were transfected with an increasing dose of Flag-HSPA8 plasmid for 24 h and then infected with ASFV (MOI = 1) for another 24 h. Viral replication was analyzed by western blotting (**R**) and RT-qPCR (**S**). Data are presented as the means ± SDs of three independent experiments and analyzed using Student’s *t*-test. *, *P* < 0.05; **, *P* < 0.01; ***, *P* < 0.001.

LDs can release fatty acids to promote the replication of several viruses. Fatty acids are primarily released from LDs through lipolysis and lipophagy. Lipolysis is catalyzed by adipose triglyceride lipase (ATGL), hormone-sensitive lipase (HSL), and monoacylglycerol lipase (MGL) (39). We analyzed the expression levels of ATGL and HSL proteins. Although ATGL expression remained unchanged, HSL was significantly upregulated during ASFV infection (Fig. 6A). To explore the role of lipophagy in ASFV infection, the protein levels of key autophagy protein, LC3, were detected. Western blotting analysis exhibited significant accumulation of LC3-I and a decrease in LC3-II during the middle and late stages of infection, indicating that autophagy was suppressed at these stages (Fig. 6B). These data demonstrate that lipolysis may be the primary mechanism by which ASFV utilizes LDs.

We reasoned that for lipolysis to proceed efficiently, the ASFV must overcome the intrinsic protective mechanisms of LDs. Previous studies have established that PLIN2 serves as a critical barrier that protects LDs from lipase-mediated degradation (40). Since our data indicated that ATGL expression remained unchanged, but PLIN2 decreased during ASFV infection. RT-qPCR analysis also showed that PLIN2 mRNA levels remained unchanged during ASFV infection, indicating that the reduction in PLIN2 is mediated at the protein level (Fig. 6C). Therefore, we hypothesize that ASFV might promote lipolysis through degraded PLIN2. Since PLIN2 has been reported to be degraded via chaperone-mediated autophagy (CMA) to facilitate lipolysis (41), we investigated whether ASFV hijacks this specific pathway. Western blotting analysis exhibited PLIN2 protein levels were restored when ASFV replication was inhibited, confirming that PLIN2 degradation is virus-dependent (Fig. 6D and E). It has been reported that PLIN2 is recognized by HSPA8, which mediates PLIN2 degradation via CMA to facilitate lipolysis (38). We purified LDs from ASFV-infected PAMs and found that HSPA8 was enriched in purified LDs (Fig. 6F). Importantly, treatment of ASFV-infected PAMs with the lysosomal inhibitor NH_4_CL almost completely restored PLIN2 degradation (Fig. 6G), and immunofluorescence analysis confirmed HSPA8 interaction with LDs during ASFV infection (Fig. 6H and I). These data showed that PLIN2 degradation during ASFV infection may be mediated by HSPA8 enrichment in LDs. To further explore the effect of CMA on PLIN2 expression, PLIN2 was co-transfected with HSPA8 and/or LAMP2A. Western blotting analysis showed that overexpression of HSPA8 and/or LAMP2A mediated PLIN2 degradation (Fig. 6J). Additionally, Co-IP analysis confirmed the interaction between HSPA8 and PLIN2 (Fig. 6K). These data indicate that ASFV infection degrades PLIN2 via CMA. To verify the role of PLIN2 degradation in the replication process of ASFV, we knocked down PLIN2 in PAMs, followed by ASFV replication. Western blotting and RT-qPCR analysis showed that knockdown effectively promoted ASFV replication (Fig. 6L and M). Furthermore, we overexpressed PLIN2 separately in WSL cells, followed by ASFV infection. Analysis of ASFV replication revealed that PLIN2 overexpression significantly suppressed ASFV replication (Fig. 6N and O). Meanwhile, PLIN2 was partially restored, and ASFV replication was inhibited when HSPA8 was knocked down in PAMs (Fig. 6P and Q), whereas HSPA8 overexpression enhanced ASFV replication (Fig. 6R and S). This indicates that reduced PLIN2 protein abundance favors ASFV replication. Collectively, these data demonstrate that ASFV manipulates CMA to degrade PLIN2, thereby promoting viral replication.

### Fatty acids of LDs were transferred to mitochondria through LD-mitochondrion contact

**Fig 7:**
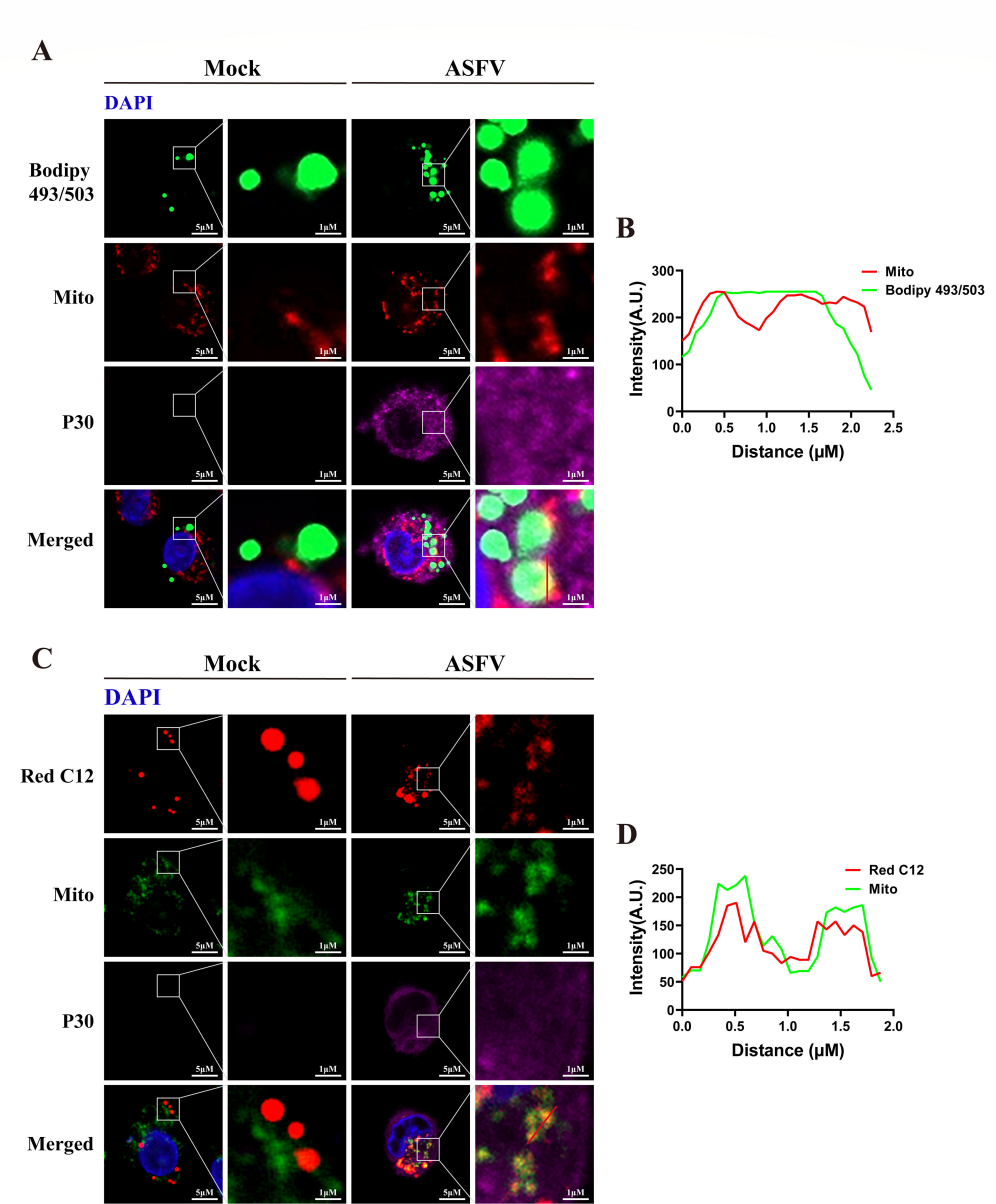
LDs release fatty acids to mitochondria through LD-mitochondrion interaction during ASFV infection. (**A and B**) ASFV infection induces LD-mitochondrion interaction in PAMs. PAMs were infected with ASFV (MOI = 0.1) or mock-infected for 24 h and then fixed and stained with anti-p30 (purple). LDs were labeled with Bodipy 493/503 (green), mitochondria were visualized with MitoMarker Red CMXRos (red), and the nucleus was stained with DAPI (blue). Cells were imaged by confocal microscopy (**A**). Scale bars, 5 μm. Intensity within the indicated area was measured (**B**). (**C and D**) LDs release fatty acids to mitochondria during ASFV infection. PAMs were treated with BODIPY 558/568 C12 for 16 h and then infected with ASFV (MOI = 1) or mock-infected for 8 h. Mitochondria were visualized with Mito-Tracker Green (green). After fixation, the PAMs were stained with anti-p30 (purple), and the nucleus was labeled with DAPI (blue). Cells were imaged by confocal microscopy (**C**). Scale bars, 5 μm. Intensity within the indicated area was measured (**D**).

LDs can dynamically interact with other membrane-bound organelles (42). Upon energy stress, fatty acids are transferred from LDs to mitochondria to generate ATP through LD-mitochondrion contacts, thereby avoiding lipid toxicity (43, 44). We explored whether LDs interact with mitochondria during ASFV infection. Confocal microscopy revealed that ASFV infection induced LD-mitochondrion tethering (Fig. 7A and B). To investigate whether LD-mitochondrion interactions mediate fatty acid transfer to mitochondria, we performed a pulse-chase assay using BODIPY 558/568-C12 (Red C12), a fluorescent fatty acid analog that accumulates in neutral lipids within LDs (43). PAMs were treated with 1 µM Red C12 overnight, washed, and infected with ASFV for 8 h. Compared to mock-infected PAMs, Red C12 was transferred to mitochondria under ASFV infection (Fig. 7C and D). These results show that ASFV infection induces LD-mitochondrion tethering to promote fatty acid transfer from LDs to mitochondria.

## DISCUSSION

**Fig 8:**
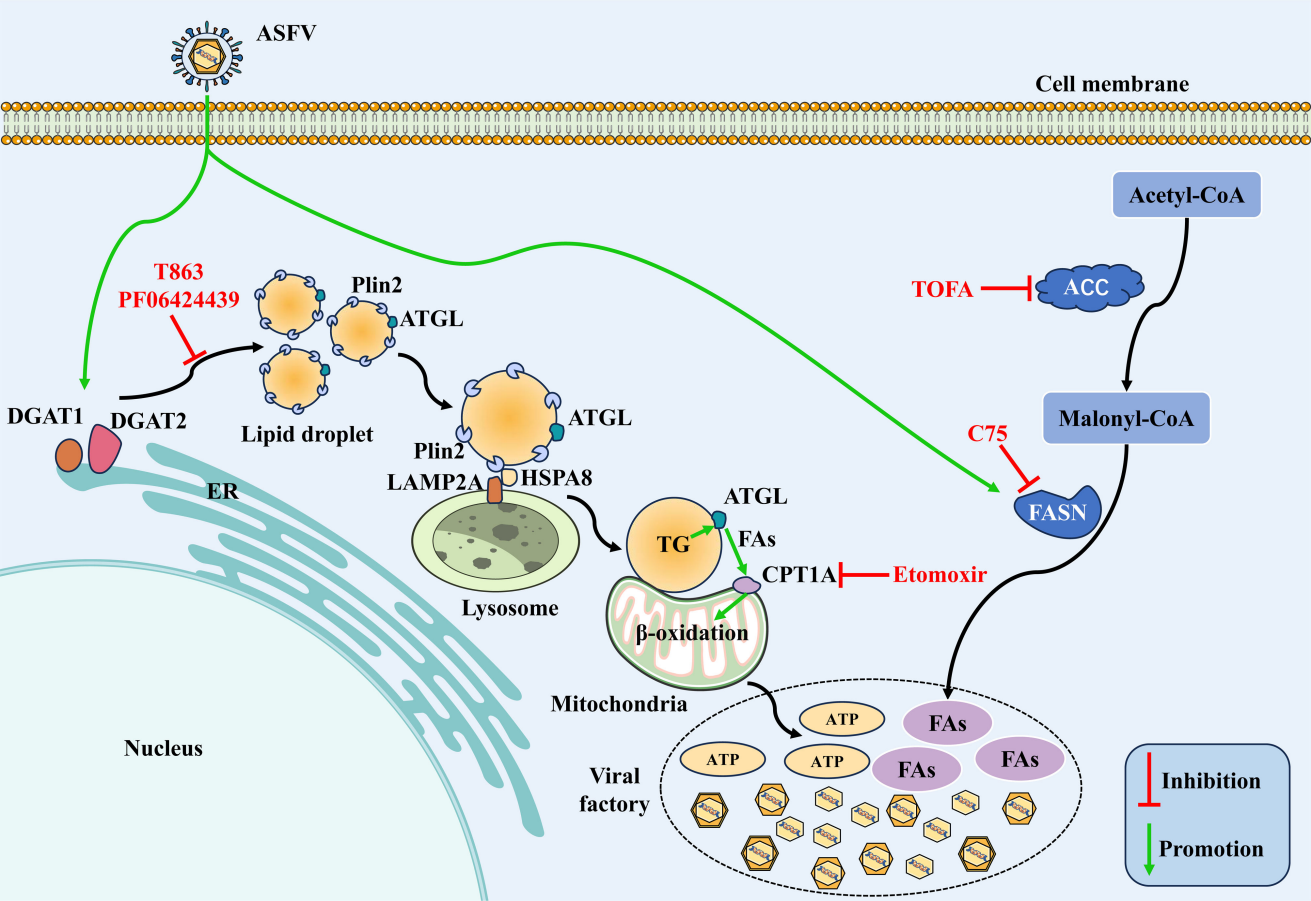
Schematic diagram of the mechanism of ASFV-induced fatty acid synthesis upregulation and CMA-mediated lipolysis to promote viral replication. Upon invading macrophages, ASFV enhances the expression of FASN to upregulate fatty acid synthesis, thereby providing fatty acids for viral replication. ASFV infection also increases the expression of DGAT1 and DGAT2 to promote LD biogenesis. Importantly, ASFV targets the LD-stabilizing protein PLIN2 for degradation via CMA, which releases stored fatty acids. These liberated fatty acids are transported to mitochondria through LD-mitochondrion interactions, ultimately promoting FAO and providing energy for viral replication.

Numerous pathogens have evolved specific mechanisms to reprogram host lipid metabolism, creating favorable conditions for replication (36, 45). In this study, we uncover a sophisticated mechanism by which ASFV exploits host lipid metabolism, particularly LD dynamics, to fuel its replication. We demonstrate that ASFV simultaneously upregulates both FAS and FAO, with LDs serving as a crucial metabolic hub that coordinates these seemingly opposing pathways by supplying fatty acids to FAO (Fig. 8). Mechanistically, ASFV increases the protein levels of FASN to upregulate FAS. Meanwhile, ASFV degrades PLIN2 via CMA, thereby releasing stored fatty acids. These liberated fatty acids are then shuttled to mitochondria through LD-mitochondrion interactions, ultimately promoting FAO to provide energy for viral replication. This intricate metabolic reprogramming ensures a continuous supply of both structural lipids and ATP for ASFV replication, highlighting a novel strategy employed by ASFV to hijack host metabolic homeostasis.

Under normal physiological conditions, FAS and FAO maintain a mutual restriction relationship. Malonyl-CoA, a critical FAS intermediate, inhibits CPT1A-mediated FAO, establishing a dynamic reciprocal regulation between these two opposing metabolic pathways (46). HCMV and HCV upregulate FAS and downregulate FAO, resulting in fatty acid accumulation in the cytoplasm for viral replication (12, 47–49). In contrast, the strategy of ASFV hijacking lipid metabolism is similar to that of other viruses, such as DENV and vaccinia virus, which exploit lipid synthesis and catabolism to support replication (10, 28, 50). However, we provide the first comprehensive dissection of this bidirectional regulatory mechanism in the context of our study. Our data demonstrate that ASFV increases the expression of FASN and CPT1A to upregulate FAS and FAO. Fatty acids synthesized via FAS maybe can stored in LDs, serving as building blocks for viral membrane synthesis. Enhanced FAO provides ATP to meet the energy-intensive needs of viral replication processes. Pharmacological inhibition of FAS (via C75/TOFA) or FAO (via etomoxir) significantly impaired viral replication, underscoring the importance of these pathways during ASFV infection. This dual upregulation likely reflects that ASFV requires sufficient lipids for structural assembly and energy for viral replication.

LDs are important organelles for maintaining lipid homeostasis in cells. In recent years, LDs have become increasingly recognized as central hubs in viral infections, serving as platforms for replication, assembly, or energy production. The SARS-CoV-2 NSP6 protein interacts with LDs to build double-membrane vesicles, enabling RNA replication (51). LDs serve as platforms for HCV through the nucleocapsid core, recruiting viral replication complexes to LDs (25). DENV and SARS-CoV-2 utilize LDs in different ways to provide energy for the viral replication process (26, 28). However, the role of LDs in ASFV infection remains largely unknown. In this study, lipidomic analysis showed that TAGs and DAGs were the most dynamically altered metabolites during ASFV infection. We also observed that ASFV induced LD accumulation in PAMs. Furthermore, inhibitors of DGAT1 and DGAT2 significantly suppressed ASFV replication, while OA promoted LD accumulation, enhancing viral replication. These findings indicated that LDs positively regulate ASFV production. Additionally, FFAs were significantly increased in PAMs after ASFV infection. Previous studies have shown that LDs can be broken down to release fatty acids to promote viral replication through lipolysis or lipophagy (52). We found that ASFV infection does not change the expression of the key enzyme of lipolysis, ATGL, and autophagy is inhibited in the middle and late stages of ASFV infection. Notably, the mRNA level of PLIN2 remained unchanged, while its protein level decreased after ASFV infection, indicating that ASFV degrades PLIN2 in some way. PLIN2 is a constitutive LD protein that protects LDs from lipolysis. Our data showed that HSPA8 is enriched in the surface of LD during ASFV infection, and the lysosomal inhibitor NH_4_Cl restored PLIN2 degradation and inhibited ASFV replication. Additionally, PLIN2 interacts with HSPA8, and PLIN2 is degraded by the overexpression of HSPA8 and LAMP2A. These data revealed that ASFV degrades PLIN2 through CMA to promote viral replication. Although our data strongly support CMA as the principal mechanism for PLIN2 degradation during ASFV infection, we cannot formally rule out the contribution of direct viral modification of PLIN2, which needs further exploration. PLIN2 has been reported to interact with ATGL to inhibit its enzymatic activity (53). The degradation of PLIN2 potentially impairs the inhibitory effect of PLIN2 on ATGL, consistent with the upregulation of hormone-sensitive lipase (HSL) during ASFV infection. Thus, these findings support that ASFV promotes lipolysis through CMA-mediated degradation of PLIN2. Given that LD biogenesis occurs at the endoplasmic reticulum (ER), where many ASFV regulatory proteins, such as p17 and EP84R, are also localized (42, 54, 55). This co-localization prompts us to speculate that these viral proteins may directly or indirectly regulate LD metabolism. Notably, previous research has reported that p17 interacts with HSPA8, which further supports the hypothesis that p17 regulates CMA to facilitate the lipolysis of LD (56). Future research will focus on screening these ER-localized viral proteins to elucidate their specific effects on LD biogenesis and lipolysis.

Fatty acids released from LDs can transfer to mitochondria through LD–mitochondrion contacts. The dynamic interaction between LDs and mitochondria has been reported in tumor cells and viral infections (26, 37). It has been shown that mitochondrial attachment to LDs promotes the trafficking of fatty acids to mitochondria to avoid lipid toxicity caused by the overabundance of fatty acids in the cytoplasm (57). We demonstrate that ASFV induces LD-mitochondrion interactions, and fatty acids stored in LDs are transferred to mitochondria. BODIPY-C12 tracing experiments provided visual confirmation of this metabolic coupling, demonstrating fatty acid flux from LDs to mitochondria and subsequent FAO in ASFV-infected PAMs. Although the specific mechanism by which ASFV induces LD-mitochondrial interactions remains unclear, these findings still demonstrate that LDs act as the main lipid source for FAO during ASFV infection and explain the simultaneous upregulation of FAS and FAO during ASFV infection. ASFV aggressively consumes free fatty acids, acetyl-CoA, and ATP. This likely creates local nutrient theft, starving immune cells of the resources needed to mount an effective response. Therefore, we hypothesize that the metabolic reprogramming we describe may be a dual-purpose strategy, fulfilling both the bioenergetic demands of viral replication and suppressing host antiviral immunity. This paradigm opens a vital avenue for future research.

In summary, our study indicates that ASFV reprograms host lipid metabolism by integrating FAS, LD biogenesis, CMA-mediated lipolysis, and FAO. ASFV upregulates both FAS and FAO, creating a favorable environment for viral replication. Importantly, ASFV mobilizes LDs through CMA-mediated lipolysis and mediates the flow of fatty acids within LDs to mitochondria via LD-mitochondrion contacts. These findings highlight the critical role of lipid metabolic reprogramming in ASFV replication and pathogenesis, which points to potential strategies for blocking viral production by targeting lipid metabolism.

## MATERIALS AND METHODS

### Cells and viruses

Porcine alveolar macrophages (PAMs) were isolated from 4-week-old specific-pathogen-free (SPF) piglets following an established protocol described previously (58) and cultured in Roswell Park Memorial Institute 1640 medium containing 10% fetal bovine serum (FBS; Excell, FSP500), 100 units/mL penicillin, 100 μg/mL streptomycin, and 25 ng/mL amphotericin B (Solarbio, P7630). Wild boar lung (WSL) and HEK-293T cells were maintained in Dulbecco’s modified Eagle’s medium (DMEM) supplemented with 10% FBS containing 100 units/mL penicillin, 100 μg/mL streptomycin, and 25 ng/mL amphotericin B. All cell cultures were incubated at 37°C with 5% CO_2_. The ASFV CN/GS/2018，ASFV-GFP and ASFV-RFP strains were provided by Lanzhou Veterinary Research Institute, the Chinese Academy of Agricultural Sciences.

### Animal infection and sample collection

Animal experiments were performed in enhanced biosafety level 3 facilities at LVRI of CAAS. According to the Animal Ethics Procedures and Guidelines of the People’s Republic of China, these experiments were conducted strictly with good animal practice. This study was approved by the Animal Ethics Committee of LVRI of CAAS.

Landrace pigs (age, approximately 75 days; weight, 25–30 kg; *n* = 4), which were free of porcine reproductive and respiratory syndrome virus (PRRSV), pseudorabies virus (PRV), porcine epidemic diarrhea virus (PEDV), and porcine circovirus type 2 (PCV-2), were obtained from a high-health farm. Pigs were randomly categorized into two groups (two pigs infected with 1 HAD_50_ of ASFV CN/GS/2018 and two pigs injected with an equal volume of PBS). Lung and spleen samples were collected from ASFV-infected pigs immediately after euthanasia in the moribund stage, and the lung and spleen samples of mock-infected pigs were collected simultaneously. Then, frozen via immersion in liquid nitrogen and stored at −80°C.

### Antibodies and reagents

The antibodies anti-HSPA8 (10654-1-AP), anti-HSL (17333-1-AP), anti-ATGL (55190-1-AP), anti-CPT1A (66039-1-Ig), anti-LC3 (14600-1-AP), anti-Flag (66008-4-Ig), anti-HA (51064-2-AP), anti-V5 (14440-1-AP), and anti-β-actin (66009-1-lg) were purchased from Proteintech; anti-PLIN2 (A24464) was purchased from Abclonal; anti-FASN (EPR7466) was purchased from Abcam; Horseradish peroxidase (HRP)-conjugated goat anti-mouse IgG (A21010) and anti-rabbit IgG (A21020) antibodies were purchased from Abbkine; anti-mouse IgG antibodies labeled with Alexa Fluor 568 (A11004) and anti-mouse IgG antibodies labeled Alexa Fluor Plus 647 (A32733) were purchased from Thermo Fisher Scientific; and anti-p72 and anti-p30 were prepared in our laboratory. The antibodies described above were used at dilutions of 1:500 for immunofluorescence and 1:1,000 for immunoblotting analysis. TOFA (S6690), C75 (S9819), and Etomoxir (E4787) were purchased from Selleck. Palmitic acid-BSA (Kc003) and oleic acid-BSA (Kc005) were purchased from Kunchuang Technology. NH_4_Cl (HY-Y1269), PF-06424439 (HY-108341), T863 (HY-32219), BODIPY 493/503 (HY-W090090), and BODIPY 558/568 C12 (HY-138226) were purchased from MedChemExpress. MitoMarker Red CMXRos (CS326) was purchased from ZFdows Bio. Mito-Tracker Green (C1048) and 4′,6-diamidino-2-phenylindole (C1002) were purchased from Byotime.

### Plasmid transfection and RNA interference

The cDNAs encoding various swine proteins were amplified by PCR from cDNAs of PAMs. HA-tagged PLIN2, Flag-tagged HSPA8, and V5-tagged LAMP2A were cloned into the pCDNA3.1 vector. All plasmids were introduced into WSL or HEK293T cells using the polyplus transfection reagent (jetPRIME, 101000046), according to the manufacturer’s instructions. The siRNAs used in this study were synthesized by Tsingke Biotechnology. PAMs were seeded in 12-well plates, and transfections were performed with the Lipofectamine RNAiMAX reagent (Invitrogen, 13778500), according to the manufacturer’s instructions. The medium was replaced with RPMI 1640 containing 10% FBS for subsequent culture. The knockdown efficacy of each protein was assessed using an immunoblot. The siRNAs targeting *FASN*, *CPT1A, PLIN2* and *HSPA8* were as follows: FASN: 5′-AUGUUCGACUUGGUGGAUCTT-3′, CPT1A: 5′-GGGAAAUCGAGCAGCAGAUTT3', PLIN2: 5'- GAGCAUAUAGAGUCACGUATT-3′, and HSPA8: 5′-GCUGUUGUCCAGUCUGAUATT-3′.

### Cell viability assay

To evaluate the cytotoxicity of inhibitors and metabolites used in this study on PAMs, Cell Counting Kit-8 (Abbkine, KTA1020) was used according to the manufacturer’s instructions. Briefly, the PAMs were seeded in 96-well plates for 4 h, followed by being treated with the indicated drugs at the indicated concentrations for 24 h. CCK-8 solution was added to each well and incubated at 37°C for an additional 1 h. The absorbance at 450 nm was measured using a microplate reader (BioTek Synergy HTX, Agilent).

### Immunoblotting analysis

The protein samples were denatured by heating at 100°C for 10 min and then were electrophoresed on 10% or 8% SDS-PAGE gels, depending on the molecular weight of the relevant proteins, and transferred to NC membranes (EMD Millipore, Billerica, MA, USA). Membranes were blocked with 5% skim milk at room temperature for 1 h. After washing three times with Tris-buffered saline–Tween 20 (TBST; containing 0.1% Tween 20), the membranes were incubated with the relevant primary antibodies overnight at 4°C. The HRP-conjugated secondary antibody was incubated at room temperature for 1 h after three times washing. Finally, the results were observed using the electrochemiluminescence solution (K-12045-D50, Advansta), and images were acquired from the Bio-Rad imaging system.

### Immunofluorescence analysis

PAMs grown on specialized confocal imaging dishes (801001, Nest) were fixed with 4% paraformaldehyde for 30 min, permeabilized with 0.2% Triton X-100 for 10 min, and blocked with 5% BSA for 1 h. The cells were then incubated with primary antibodies in 1% BSA overnight at 4°C and incubated with secondary antibodies for 1 h at room temperature. LDs were stained with BODIPY 493/503 in PBS for another 30 min at room temperature. Finally, the cells were stained with 4′,6-diamidino-2-phenylindole (DAPI) for 10 min. Images were captured using a laser scanning confocal microscope (LSM980, Zeiss) and processed with ImageJ software for quantitative image analysis.

#### Real-time qPCR

Total RNA was extracted using TRIzol Reagent (15596018CN, Thermo Fisher Scientific) and reverse-transcribed into cDNA through a PrimeScript RT Reagent Kit (RK20433, Abclonal). The RT-qPCR was performed in triplicate by using SYBR Premix Ex Taq (RK21219, Abclonal) on the ABI StepOnePlus system. The data were normalized to the level of GAPDH expression in each sample. The relative expression change of each target gene was calculated using the 2^−ΔΔCT^ method. Primers used for RT-qPCR are listed in Table 1.

**TABLE 1:** List of primers used in the RT-qPCR analysis

Primers	Sequence (5′→3′)
PLIN2-qF	GCAACAGAGGCAAAAGAATGA
PLIN2-qR	CAAGTGGAGAAGCAGCTAGTG
DGAT1-qF	TGGACTACTCACGCATCAT
DGAT1-qR	GTGGAAGAGCCAGTAGAAGAA
DGAT2-qF	GCGGGAGTACCTGATGTCTG
DGAT2-qR	AACCAGGTCGGCTCCGT
p72-qF	TGCGATGATGATTACCTT
p72-qR	ATTCTCTTGCTCTGGATAC
GAPDH-qF	ACATGGCCTCCAAGGAGTAAGA
GAPDH-qR	GATCGAGTTGGGGCTGTGACT

ASFV genomic DNA was extracted from cells using the QIAamp DNA Mini Kits (Qiagen, Germany). Subsequently, the copy number of the ASFV genome was detected as previously described (59). qPCR was performed using Pro Taq HS Premix Probe qPCR kit (Accurate Biology, China) via the ABI StepOnePlus system. The TaqMan probe and p72 primers used for qPCR were as follows: p72-F, 5′-GATACCACAAGATCAGCCGT-3′; p72-R, 5′-CTGCTCATGTATCAATCTTATCGA-3′; and TaqMan, 5′-CCACGGGAGGAATACCAACCCAGTG-3′.

### Lipid extraction

To explore the change of lipid after ASFV infection, PAMs were infected with ASFV (MOI = 0.1) at different time points (0, 6, 12, 24, and 48 h). Following infection, the cells were harvested and washed with PBS. The samples were then mixed with 250 μL of water, followed by vortexing for 60 s, freezing and thawing in liquid nitrogen three times, and sonication for 20 min in an ice-water bath. A 50 μL aliquot of the homogenate was taken for BCA quantification. For lipid extraction, 200 μL of the homogenate was combined with 480 μL of an extraction solution (MTBE:MeOH = 5:1) containing an internal standard. After a 60 s vortex, the samples were sonicated for 10 min in an ice-water bath and then centrifuged at 3,000 rpm for 15 min at 4°C; 250 μL of the supernatants was carefully transferred to a new tube. The remaining sample was treated with an additional 250 μL of MTBE, and the process of vortexing, sonication, and centrifugation was repeated to ensure thorough extraction. This step was conducted twice more. The collected supernatants were pooled and evaporated to dryness using a vacuum concentrator at 37°C. Then, the dried samples were reconstituted in 100 μL of resuspension buffer (DCM:MeOH:H_2_O = 60:30:4.5), and the samples were vortexed for 30 s and sonicated for 10 min in an ice-water bath. The constitution was then centrifuged at 12,000 × *g* for 15 min at 4°C, and 35 μL of the supernatant was then transferred to a fresh glass vial for LC/MS analysis. The quality control (QC) sample was prepared by mixing an equal aliquot of the supernatants from all of the samples.

### Liquid chromatography MS/MS analysis

The ultra-high-performance liquid chromatography (UHPLC) separation was carried out using a Nexera LC-40 series UHPLC System. The mobile phase A consisted of 40% water, 60% acetonitrile, and 10 mmol/L ammonium formate. The mobile phase B consisted of 10% acetonitrile, 90% isopropanol, and 10 mmol/L ammonium formate. The column temperature was 45°C. The auto-sampler was set to 10°C. The injection volume for each sample was 2 μL. A SCIEX Triple Quad 7500 mass spectrometer was applied for assay development. Typical ion source parameters were as follows: ion spray voltage, +3,500/−3,000 V; curtain gas, 50 psi; temperature, 400°C; ion source gas 1, 50 psi; and ion source gas 2, 50 psi. Prior to mass spectrometry analysis, the most suitable Q1/Q3 transition pair was selected for each target lipid, and the multiple reaction monitoring (MRM) parameters were fine-tuned to ensure optimal sensitivity and selectivity. These ion pairs were then utilized for both qualitative and quantitative analysis.

### Co-immunoprecipitation

HEK-293T cells were transfected with the indicated plasmids for 24 h and collected in cold PBS and centrifuged at 500 × *g* for 10 min. The supernatant was removed, and the cells were re-suspended in NP40 buffer supplemented with protease inhibitor cocktail and incubated for 30 min at 4°C. Then, the samples were centrifuged at 12,000 *× g* for 20 min at 4°C, 10% of the supernatants were saved as input samples for analysis by immunoblotting, and 50 μL of beads was washed with lysis buffer and added to the remaining protein samples and incubated overnight at 4°C. The beads were washed with lysis buffer three times before adding 1× loading buffer. Precipitated proteins were analyzed by western blotting.

### LD isolation

The LDs were isolated from the PAMs following the manufacturer’s instructions (Cell Biolabs, Inc., catalog no. MET-5011). Briefly, 1.5 × 10^7^ PAMs were washed with PBS three times, and the medium was removed. The cell pellet was resuspended with 200 µl of reagent A. After incubating for 10 min, 800 µL of reagent B was added, and the cells were homogenized by passing the cells five times through a 27-gauge needle. Then, 600 µL of the first layer of reagent B was taken and centrifuged for 3 h (18,000 × *g*, 4°C). The LDs were contained in the top 270 µL of reagent B. The distribution of PLIN2 (LD surface protein marker) was then assessed.

### Flow cytometry

To assess the alterations in LDs in the ASFV-infected cells, PAMs were cultured in six-well plates and subsequently infected with ASFV-RFP. The infected cells were harvested at 24 hpi via centrifugation at 500 × *g* for 10 min, and then stained with BODIPY 493/503 (1 µg/mL) for 30 min in PBS containing 2% (wt/vol) FBS. The cellular preparations were then analyzed by flow cytometry using a flow cytometer (Beckman, California, USA) equipped with FlowJo software to visualize LDs.

### Pulse-chase assay

PAMs were incubated in a complete medium (RPMI 1640 with 10% fetal bovine serum) containing 1 µM of BODIPY 558/568 C12 for 16 h. The treated cells were washed with PBS three times and then infected with ASFV for another 8 h.

### Viral titration

The samples containing ASFV were quantified using the hemadsorption (HAD) assay as described previously (60). PAMs were spread onto a 96-well plate. The sample was diluted to 10^−1^, 10^−2^, 10^−3^, 10^−4^, 10^−5^, 10^−6^, 10^−7^, and 10^−8^ and was added to a 96-well plate. The adsorption of erythrocytes was observed for 7 days. The 50% hemadsorption dose was calculated according to the Reed–Muench method (61).

### Statistical analysis

In lipidomics, all target metabolite analysis and quantification work are completed through BioBud (v2.0.3) software. To identify significantly altered lipids, a two-component criterion was applied. First, unpaired two-tailed Student’s *t*-tests were performed, and lipids with a *P*-value <0.05 were considered for the next step. Second, a supervised partial least squares-discriminant analysis (PLS-DA) was conducted, and the variable importance in projection (VIP) score for each lipid was extracted. Lipids were required to have a VIP score >1. A lipid was definitively classified as significantly altered only if it simultaneously met all two criteria: *P* < 0.05 and VIP > 1. All *in vitro* experiments were performed at least three times. Data are presented as the means ± standard deviations (SDs). The statistical significance between groups was determined using the unpaired two-tailed Student’s *t*-test with GraphPad Prism v.8 (San Diego, CA, USA). *, *P* < 0.05, **, *P* < 0.01, and ***, *P* < 0.001 were considered statistically significant; ns: no significant difference.

## SUPPLEMENTAL MATERIAL

Fig. S1 mbio.03368-25-s0001.tif C75 affects ASFV replication but not binding and entry.

Fig. S2 mbio.03368-25-s0002.tif Heat map of hierarchical clustering analysis of differential metabolites after ASFV infection.

Fig. S3 mbio.03368-25-s0003.tif The expression changes of key metabolic enzymes in ASFV-infected lung and spleen.

Fig. S4 mbio.03368-25-s0004.tif LD biogenesis inhibitors suppress the pro-viral replication effect mediated by OA.

Legends mbio.03368-25-s0005.docx Supplemental figure legends.

Table S1 mbio.03368-25-s0006.xlsx Differentially expressed metabolites in PAMs after ASFV infection.

ASM does not own the copyrights to Supplemental Material that may be linked to, or accessed through, an article. The authors have granted ASM a non-exclusive, world-wide license to publish the Supplemental Material files. Please contact the corresponding author directly for reuse.
