# Supplementary figures and images for: African swine fever virus hijacks lipolysis induced by chaperone-mediated autophagy to upregulate fatty acid β-oxidation and promote viral replication
Source: mBio. 2026 Mar 9;17(4):e03368-25. doi: 10.1128/mbio.03368-25 (PMC13064677; doi:10.1128/mbio.03368-25)

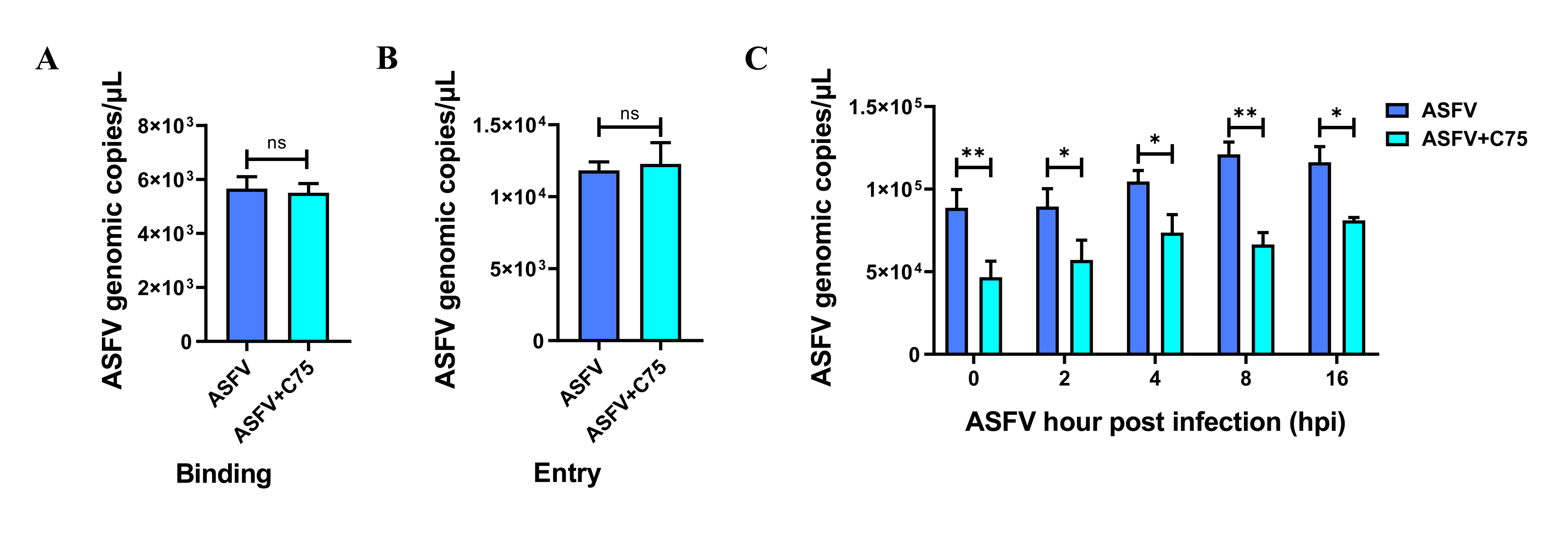

Supplement: Fig. S1 — C75 affects ASFV replication but not binding and entry. [file mbio.03368-25-s0001.tif]

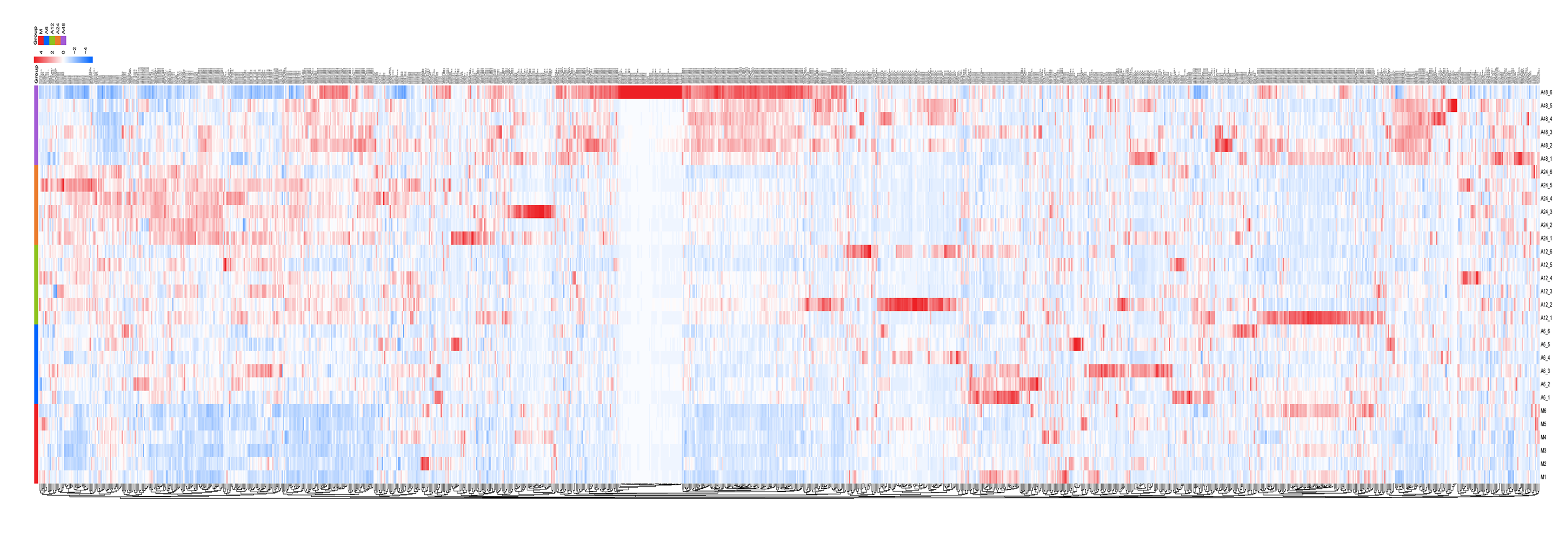

Supplement: Fig. S2 — Heat map of hierarchical clustering analysis of differential metabolites after ASFV infection. [file mbio.03368-25-s0002.tif]

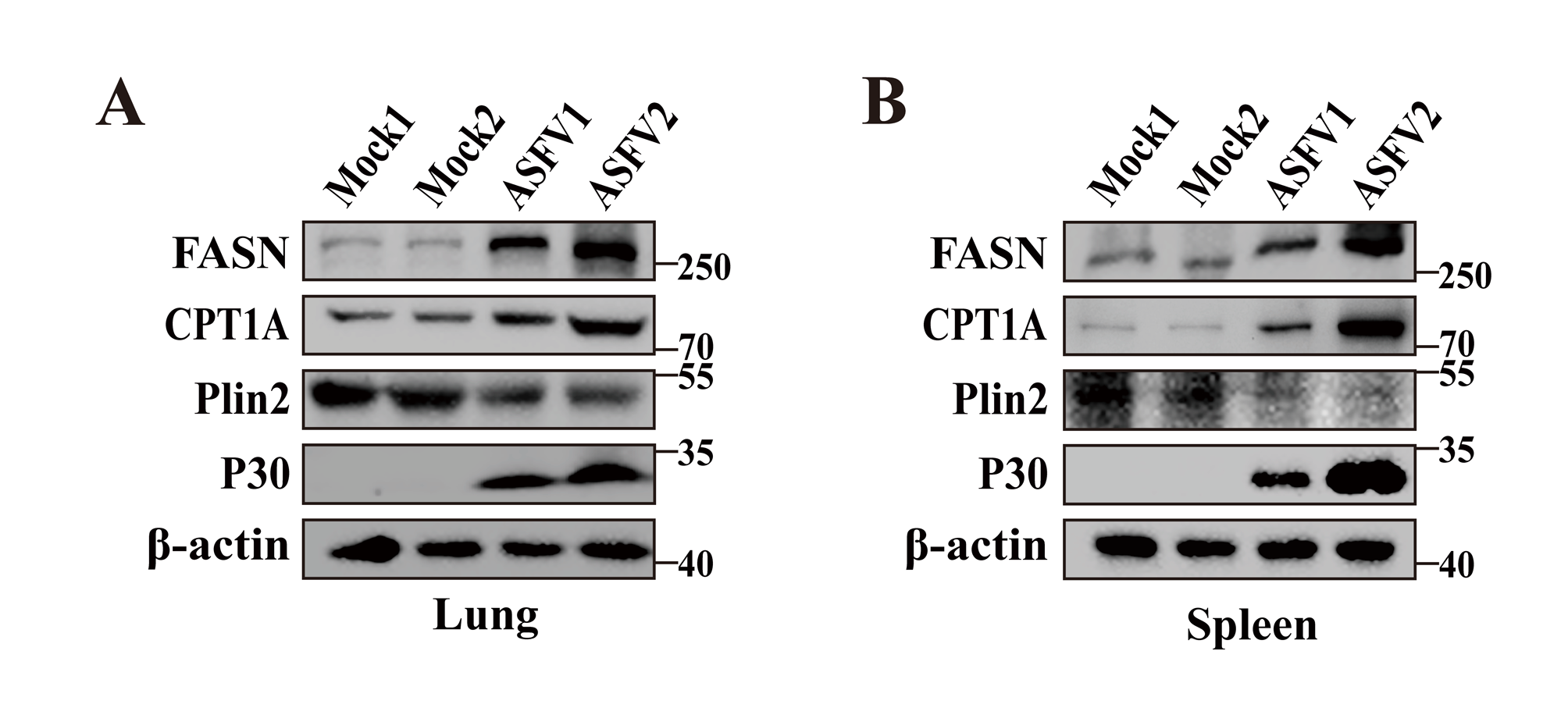

Supplement: Fig. S3 — The expression changes of key metabolic enzymes in ASFV-infected lung and spleen. [file mbio.03368-25-s0003.tif]

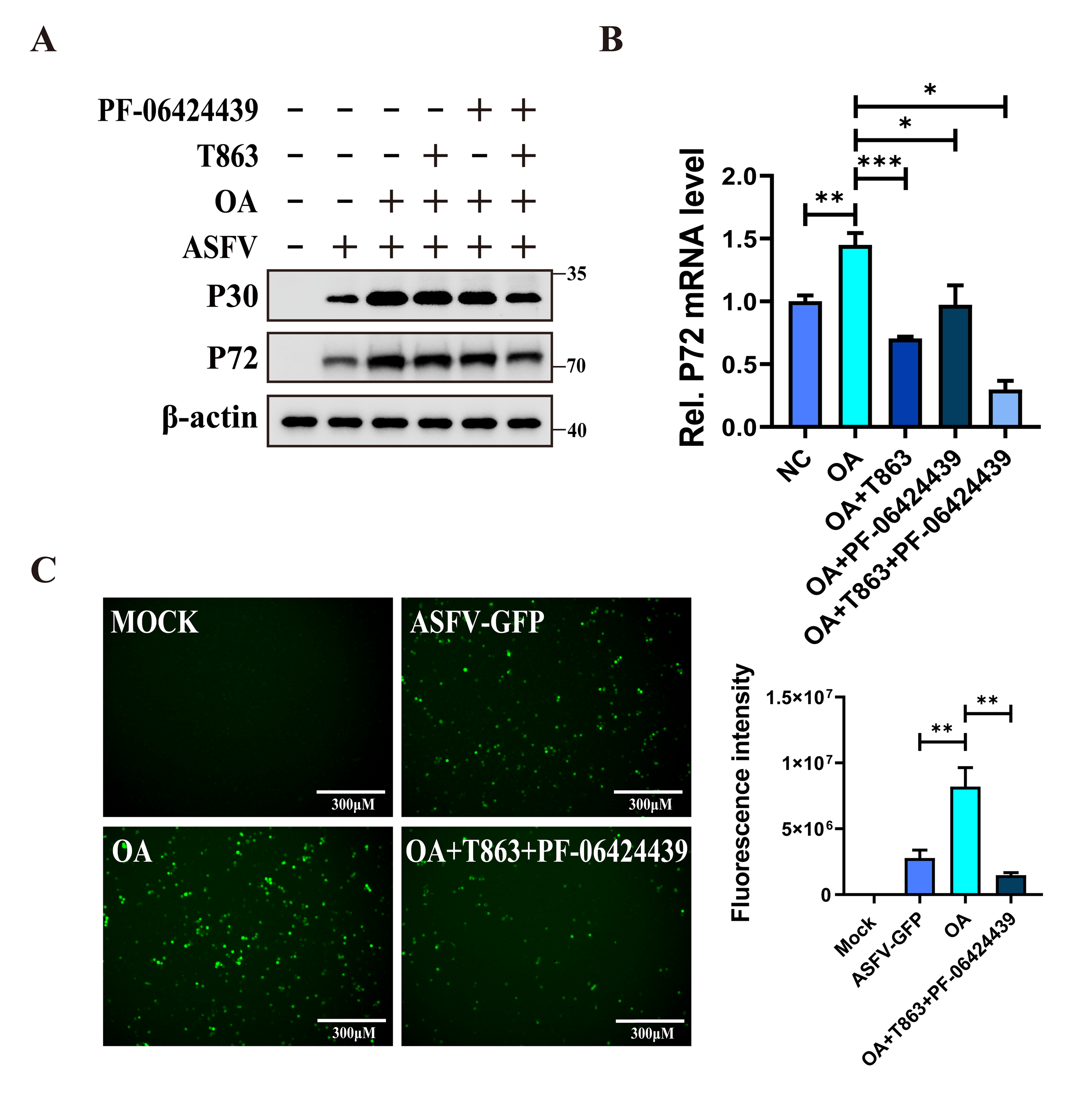

Supplement: Fig. S4 — LD biogenesis inhibitors suppress the pro-viral replication effect mediated by OA. [file mbio.03368-25-s0004.tif]
