# Supplementary material for: African swine fever virus hijacks lipolysis induced by chaperone-mediated autophagy to upregulate fatty acid β-oxidation and promote viral replication
Source: mBio. 2026 Mar 9;17(4):e03368-25. doi: 10.1128/mbio.03368-25 (PMC13064677; doi:10.1128/mbio.03368-25)
Supplement: Legends — Supplemental figure legends. [file mbio.03368-25-s0005.docx]

**Supplemental Figure Legends**

**Fig. S1 C75 affects ASFV replication but not binding and entry.**(A and B) The effects of C75 on binding and entry. C75 (25 µM) were used to PAMs and infected with ASFV (MOI =0.1) for 1 h at 4 °C and then at 37 °C at 0 hpi (binding) or 1 hpi (entry). After washing three times with PBS, PAMs continued to be cultured for 24 h. The viral genome copies were detected by qPCR. (C) Time points of addition of C75 in ASFV infection. PAMs were infected with ASFV at an MOI of 0.1 and treated with C75 (25 μM) at different time points (0, 2, 4, 8, 12, and 16 hpi). The viral genome copies were determined by qPCR at 24 hpi. Data are presented as the means ± SDs of three independent experiments and analyzed using Student's *t*-test. *, *P* < 0.05; **, *P* < 0.01; ns, not significant.

**Fig. S2 Heat map of hierarchical clustering analysis of differential metabolites after ASFV infection.**

**Fig. S3 The expression changes of key metabolic enzymes in ASFV-infected lung and spleen.** (A and B) Lung and spleen samples were collected from ASFV-infected pigs immediately after euthanasia in the moribund stage; PLIN2, FASN and CPT1A of Lung (A) and spleen (B) were analyzed by Western blotting.

**Fig. S4 LD biogenesis inhibitors** **suppress the pro-viral replication effect mediated by OA.**

(A and B) PF-06424439 or/and T863 inhibited the promotion of ASFV replication by OA. PAMs were treated with PF-06424439 (50 µM), T863 (50 µM), or both for 12 h in the presence of OA (80 µM). The same volume of DMSO was used as a control. The cells were then infected with ASFV (MOI = 0.1) for another 24 h. The expression levels of the viral proteins p30 and p72, as well as the mRNA level of p72, were analyzed by Western blotting (A) and RT-qPCR (B), respectively. (C) PF-06424439 and T863 inhibited the promotion of ASFV-GFP replication by OA. PAMs were treated with PF-06424439 (50 µM), T863 (50 µM), or both for 12 h in the presence of OA (80 µM). The same volume DMSO was used as a control. The cells were then infected with ASFV-GFP (MOI = 0.1) for another 24 h. the GFP expression were captured by a fluorescence microscope, and GFP fluorescence intensity was quantified using the Image J software. Scale bars = 300 μM. Data are presented as the means ± SDs of three independent experiments and analyzed using Student's *t*-test. *, *P* < 0.05; **, *P* < 0.01; ***, *P* < 0.001.
